# Resolving the zinc binding capacity of honey bee vitellogenin and locating its putative binding sites

**DOI:** 10.1111/imb.12807

**Published:** 2022-08-23

**Authors:** Vilde Leipart, Øyvind Enger, Diana Cornelia Turcu, Olena Dobrovolska, Finn Drabløs, Øyvind Halskau, Gro V. Amdam

**Affiliations:** ^1^ Faculty of Environmental Sciences and Natural Resource Management Norwegian University of Life Sciences Aas Norway; ^2^ Department of Biological Sciences University of Bergen Bergen Norway; ^3^ Department of Clinical and Molecular Medicine, Faculty of Medicine and Health Sciences NTNU – Norwegian University of Science and Technology Trondheim Norway; ^4^ School of Life Sciences Arizona State University Tempe Arizona USA

**Keywords:** honey bees, insect immunity, protein structure analysis, vitellogenin, zinc‐binding

## Abstract

The protein vitellogenin (Vg) plays a central role in lipid transportation in most egg‐laying animals. High Vg levels correlate with stress resistance and lifespan potential in honey bees (*Apis mellifera*). Vg is the primary circulating zinc‐carrying protein in honey bees. Zinc is an essential metal ion in numerous biological processes, including the function and structure of many proteins. Measurements of Zn^2+^ suggest a variable number of ions per Vg molecule in different animal species, but the molecular implications of zinc‐binding by this protein are not well‐understood. We used inductively coupled plasma mass spectrometry to determine that, on average, each honey bee Vg molecule binds 3 Zn^2+^‐ions. Our full‐length protein structure and sequence analysis revealed seven potential zinc‐binding sites. These are located in the β‐barrel and α‐helical subdomains of the N‐terminal domain, the lipid binding site, and the cysteine‐rich C‐terminal region of unknown function. Interestingly, two potential zinc‐binding sites in the β‐barrel can support a proposed role for this structure in DNA‐binding. Overall, our findings suggest that honey bee Vg bind zinc at several functional regions, indicating that Zn^2+^‐ions are important for many of the activities of this protein. In addition to being potentially relevant for other egg‐laying species, these insights provide a platform for studies of metal ions in bee health, which is of global interest due to recent declines in pollinator numbers.

## Graphical abstract

Honey bee vitellogenin binds 3 Zn^2+^‐ions on average. Structure and sequence analysis reveals seven potential zinc‐binding sites at different functional regions. Two potential sites support a proposed role for the β‐barrel subdomain in DNA‐binding.

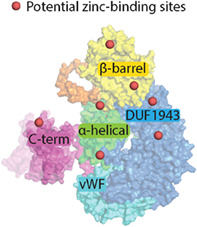

## INTRODUCTION

Zinc is necessary for living organisms to function properly (Sloup et al., 2017). The element is involved in basic life processes such as cell division and gene expression and it is an essential nutrient for growth and development (Baltaci & Yuce, 2018; Falchuk, 1998). Zinc is necessary for catalytic, structural and regulatory functions for thousands of proteins (Andreini et al., 2006). Improving the understanding of zinc‐carrying proteins is thus likely to reveal new information about many physiological processes across taxa.

An important zinc‐carrying protein in egg‐laying animals is the multi‐domain glycolipophosphoprotein vitellogenin (Vg) (Falchuk, 1998; Gupta et al., 2021; Matozzo et al., 2008; Montorzi et al., 1994). Vg provides nutrients to developing embryos by delivering lipids, amino acids and zinc (Pan et al., 1969). In some species, Vg is expressed in juveniles, as well as in males and in females that do not reproduce, hinting at roles beyond yolk formation (Sappington & Raikhel, 1998). Such roles have been most abundantly studied in honey bees (*Apis mellifera*), where Vg is recognized as a multi‐functional protein impacting the behaviour and health of workers (functionally sterile females). RNA‐interference mediated gene knockdown reveals that honey bee Vg affects worker behavioural ontogeny, foraging choice, capacity to provide larval care, stress resistance and longevity (Amdam et al., 2004; Guidugli et al., 2005). At least the two latter of the physiological impacts of honey bee Vg can be zinc‐related, as low zinc levels may reduce the cell‐based immune capacity of the workers (Amdam et al., 2005). However, apart from the finding that circulating zinc levels correlate strongly with the hemolymph level of Vg in honey bees (Amdam et al., 2004), the molecular relationship between this ion and protein is largely unknown.

The capacity for zinc‐binding is established for Vg proteins, but the number of ions per Vg molecule varies among species. For example, measurements of bound Zn^2+^‐ions in the hemolymph of shore crab (*Carcinus maenas*) (Martin & Rainbow, 1998) are higher than those of the American clawed frog (*Xenopus laevis*) (Montorzi et al., 1994) and domestic fowl (*Gallus gallus*) (Mitchell & Carlisle, 1991). The same studies also indicate that the number of Zn^2+^‐ions carried by Vg can vary with individual reproductive state and age. Such variation is likely biologically important, but to date is not well‐understood for Vg proteins.

A prerequisite for understanding zinc‐related molecular mechanisms is finding the location and structural context of Zn^2+^‐binding sites within the protein of interest (Ataie et al., 2008; Daniel & Farrell, 2014). The coordinating environment for Zn^2+^‐ions in proteins is well‐characterized (Dudev & Lim, 2003; Pace & Weerapana, 2014), and binding sites are usually sorted into two structurally distinct categories based on whether the Zn^2+^ has a catalytic or structural role. A catalytic binding site is often partially exposed to the solvent, and the Zn^2+^‐ion coordinates most often with histidine (H), cysteine (C), aspartate (D), glutamate (E), serine (S) residues and/or water molecules (Ataie et al., 2008; Jernigan et al., 1994). A structural binding site is usually buried in the protein, surrounded by an intricate network of hydrogen bonds (Dudev & Lim, 2003), and the Zn^2+^‐coordinating residues are typically multiple H/C residues only. For example, the well‐known transcription factor motif zinc fingers (C4 or C2H2) (Pace & Weerapana, 2014) represents a structural binding site.

The prevalence of coordinating residues differs between catalytic and structural binding sites. For catalytic sites, 4, 5 and 6 residues coordinate in 48%, 44% and 6% of cases, respectively. Correspondingly, the ratio is 79%, 6% and 12% for structural sites, respectively (Ataie et al., 2008; Dudev & Lim, 2003). These numbers imply that catalytic sites largely coordinate Zn^2+^ with 4 or 5 residues, while structural sites most commonly coordinate with 4 residues. In addition to these two categories, Zn^2+^‐binding is identified in regulatory Zn^2+^‐Cys complexes, called redox switches (Pace & Weerapana, 2014). Two common characteristics for all coordinating sites are: strong interaction between the residues and the Zn^2+^‐ion, and high hydrophobic contrast in the binding site (Dudev & Lim, 2003; Pace & Weerapana, 2014).

The zinc coordinating environment for honey bee Vg is not described, but speculations have been presented regarding lamprey (*Ichthyomyzon unicuspis*) and zebrafish (*Danio rerio*) Vg. Anderson et al. (1998) published the only experimentally solved protein structure of lamprey Vg (PDB‐ID: 1LSH), which lacks Zn^2+^‐ions due to use of 1 mM EDTA during crystallization. In the absence of zinc, Anderson et al. (1998) proposed two potential binding sites (H312/H322 and H868/H887) based on the residues' locations in the crystal structure and the sequence conservation. In comparison, Sullivan and Yilmaz (2018) suggest that the phosphorylated serine‐rich phosvitin domain is associated with Zn^2+^‐ions in zebrafish. This domain is missing in some Vg proteins, including those of insects (Tufail & Takeda, 2008). For example, Honey bee Vg consists of an N‐terminal domain, a lipid cavity and a C‐terminal region (Havukainen et al., 2011, 2012; Leipart et al., 2022). The N‐terminal domain comprises the β‐barrel subdomain followed by a flexible polyserine linker and the α‐helical subdomain. The lipid cavity is built up by a domain of unknown function (DUF1943), a β‐sheet, and a von Willebrand factor (vWF) domain. Honey bee Vg can be cleaved at the polyserine linker in the N‐terminal domain. This cleavage creates a small fragment (40 kDa, the β‐barrel subdomain) and a larger fragment (150 kDa, the α‐helical subdomain, the lipid binding site and the C‐terminal region) (Havukainen et al., 2012). It is interesting to note that the smaller fragment of honey bee Vg may translocate into cell nuclei, bind DNA (potentially with co‐factors) and influence gene expression (Salmela et al., 2022).

Honey bees provide a practical and useful research system as they are globally available as commercial pollinators and primary producers of honey, pollen and wax. We recently published the first full‐length Vg structure prediction for honey bees (Leipart et al., 2022) using experimental available data, computational modelling and AlphaFold (Jumper et al., 2021). The structural templates have not resolved the position of zinc, and AlphaFold does not predict the position of non‐protein components (Jumper et al., 2021). We use the model here to provide insight into possible zinc‐binding sites of Vg. First, we performed an element analysis of Vg protein obtained from worker bee hemolymph and found that, on average, it binds 3 Zn^2+^‐ions. Using our structural data in combination with sequence data, we then conducted an in‐depth analysis to predict the location(s) of potential zinc‐binding sites. We identified areas in the β‐barrel subdomain, α‐helical subdomain, lipid binding site and C‐terminal region. We propose that zinc in the β‐barrel subdomain plays a role in DNA binding. In an attempt to characterize the zinc‐binding site(s) of this subdomain, we expressed the β‐barrel using bacterial recombinant expression systems in cultural medium with various compositions of Zn^2+^ and/or Co^2+^ but this approach did not provide a clear answer. However, taken together, our results provide the first detailed insights into where honey bee Vg can bind Zn^2+^.

## RESULTS

### Identification of zinc in honey bee Vg using inductively coupled plasma mass spectrometry

**FIGURE 1:**
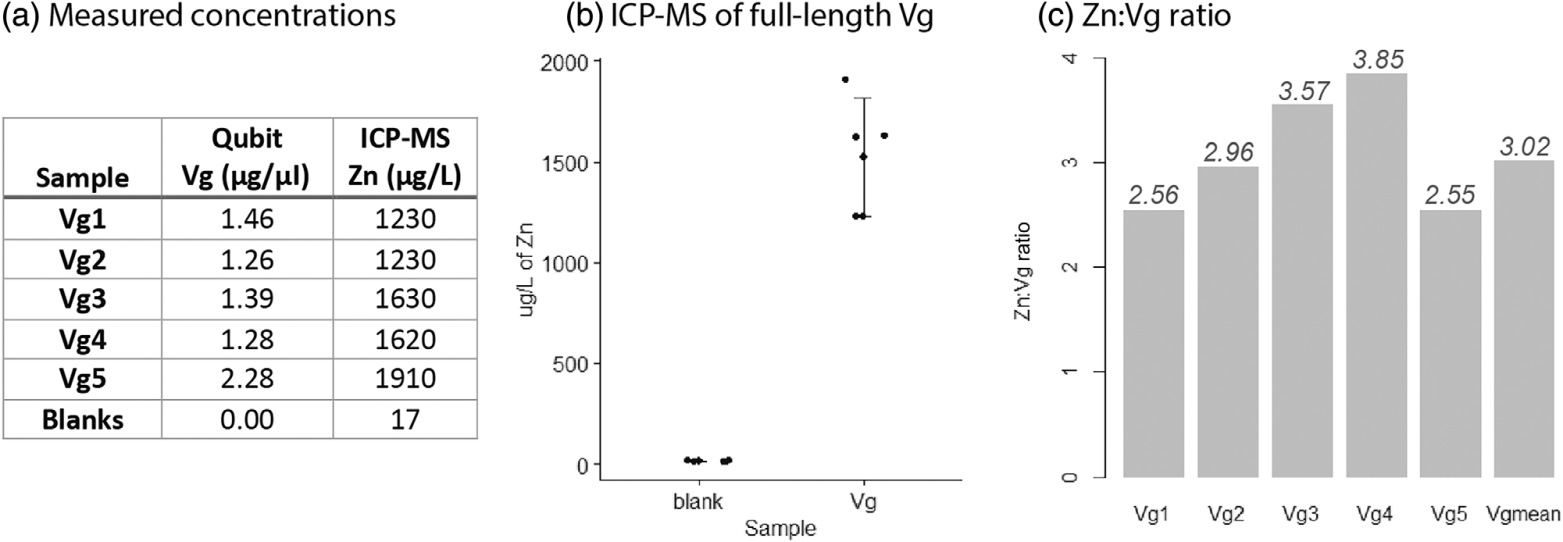
Inductively coupled plasma mass spectrometry (ICP‐MS). (a) The concentration measured with Qubit and ICP‐MS for full‐length vitellogenin (Vg) and the five samples of blank (same concentration). (b) Plot of the ICP‐MS concentration for the five blanks and full‐length Vg. The mean and the SD of the mean are indicated for each group (lines). (c) The calculated molecular Zn:Vg ratio for each sample of Vg is provided, and the calculated mean value for all samples is included as a separate bar.

We performed inductively coupled plasma mass spectrometry (ICP‐MS) on Vg from worker bee hemolymph to confirm Vg as a zinc carrier and quantify the number of Zn^2+^‐ions per Vg molecule (Figure 1a). We detected significant amounts of Zn^2+^ in Vg samples relative to the (zinc negative) controls (Kruskal–Wallis analysis: chi‐squared = 7.81, df = 1, *p*‐value = 0.00519, Figure 1a,b). Based on sample concentrations of Vg and Zn^2+^, and using the theoretical molecular weight of Vg and Zn^2+^ (201147.7 g/mol and 65.30 g/mol, respectively), we calculated the molecular Zn:Vg ratio for each sample (Figure 1c and Appendix_RatioCalculations.xlsx). This analysis gave a range of 2.55–3.85 mol of Zn^2+^‐ions per Vg molecule, with an average ratio of 3 Zn^2+^‐ions per full‐length Vg protein.

### Identification of potential zinc coordinating residues in honey bee Vg

**FIGURE 2:**
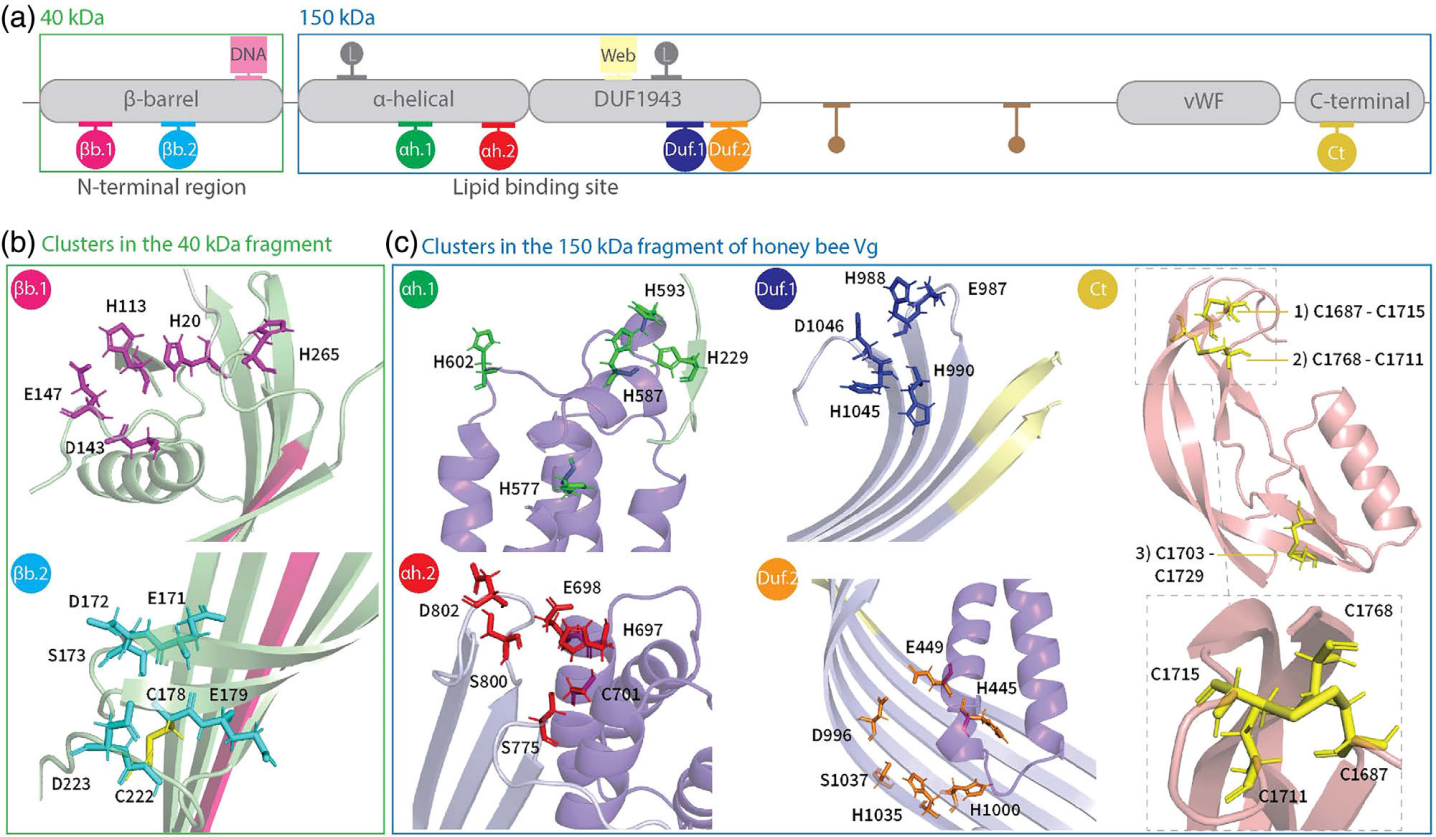
Identified clusters: (a) 2D representation of the honey bee vitellogenin (Vg) with conserved domain, subdomains and regions (grey boxes). The amino acid sequence was divided into two regions based on the naturally cleaved fragments, the α‐helical subdomain, the lipid binding site, including von Willebrand factor and C‐terminal region (blue, 150 kDa), and the β‐barrel subdomain (green, 40 kDa). The identified clusters are marked below the grey boxes (αh.1: Green, αh.2: Red, Duf.1: Blue, Duf.2: Orange, Ct: Yellow, βb.1: Pink and βb.2: Cyan). The two zinc‐binding locations identified by studies of lamprey are marked above the grey boxes with grey dots (L). The DNA binding motif (pink box, DNA) and the zinc‐binding motif identified by MotifScan (yellow box, web) are also marked above the grey boxes. Finally, the two conserved disulfide bridges not included in a cluster are included here (smaller brown dots). (b) Identified clusters in the 40 kDa fragment of honey bee Vg. The residues of cluster βb.1 (magenta) and βb.2 (cyan) are in the β‐barrel subdomain (green) and close to the DNA binding motif (pink β‐sheet). The disulfide bridge in cluster βb.2 is shown as a yellow stick. (c) Identified clusters in the 150 kDa fragment of honey bee Vg. *Cluster αh.1*: The insect‐specific loop region in the α‐helical subdomain (purple) and a short β‐strand from the β‐barrel subdomain (green) contains the residues of cluster αh.1 (green sticks). *Cluster αh.2*: The α‐helical subdomain (purple) and the β‐sheet region in the DUF1943 (light purple) are shown as a cartoon. The identified cluster‐residues are shown as red sticks. *Cluster Duf.1*: Two loops from the DUF1943 (light purple) contain the identified residues, shown as blue sticks. The zinc‐binding motif identified by MotifScan (yellow) is located in the neighbouring β‐strands in the DUF1943. *Cluster Duf.2*: Cluster Duf.2 (orange sticks) is found further down on the same β‐sheet in DUF1943 (light purple). Two of the residues are located in the α‐helical subdomain (purple). *Cluster Ct*: Three disulfide bridges (yellow sticks) are found in the C‐terminal (light pink). The dotted box zooms in on two of the disulfide bridges, showing them from another direction. The two disulfide bridges can create a tetrahedral geometry and are identified as cluster Ct.

In this section, we analyse honey bee Vg to identify zinc‐binding sites, referred to as clusters. The results are summarized in Table 1 and illustrated in Figure 2a. To achieve this, we took a comprehensive approach: using online bioinformatic tools developed for identification of zinc motifs in amino acid sequences, assessing suggested structural sites from studies on lamprey (Anderson et al., 1998) and zebrafish (Sullivan & Yilmaz, 2018), and finally, analysing our recently published full‐length protein structure (Leipart et al., 2022). We used a multiple sequence alignment (MSA) of Vg sequences with a broad phylogenetic range to evaluate the findings.

**TABLE 1:** Identified zinc clusters

Cluster	Vg domain	Residues
βb.1	β‐barrel subdomain	H20, H113, D143, E147, H265
βb.2	β‐barrel subdomain	E171, D172, S173, C178, E179, C222, D223
αh.1	α‐helical subdomain	H229, H577, H587, H593, H602
αh.2	α‐helical subdomain	H697, E698, C701, S775, S800, D802
Duf.1	DUF1943	E987, H988, H990, H1045, D1046
Duf.2	DUF1943	H445, E449, D996, H1000, H1035, S1037
Ct	C‐terminal	C1687, C1711, C1715, C1768

*Note*: The clusters are named (Column 1) based on their location in honey bee Vg (Column 2). The residues included in each cluster are listed in Column 3.

Abbreviation: Vg, vitellogenin.

#### Assessing potential zinc coordinating residues using online tools

The online motif search algorithms (see Experimental Procedures) produced only one hit. MotifScan (Pagni et al., 2007) identified a Zn^2+^‐binding motif formed by residues 926 to 936. This short region contains several conserved residues (F928, P929, G933, L934, P935 and F936, Figure S1). Regarding the MSA (Figure S1), the zinc‐binding residue identified in the motif, H926, is only present in the *A. mellifera* Vg sequence. The motif is located in a β‐strand‐turn‐β‐strand fold in the DUF1943, which extends into the β‐barrel subdomain, close to cluster Duf.1 and βb.2 and the DNA binding motif (see Figure S2 for a structural overview of this region).

#### Assessing potential zinc coordinating residues from suggested zinc‐binding sites

According to the MSA, the two sites proposed in lamprey Vg (H312/H322 and H868/H887) align to E427/V444 and N975/G994 in honey bee Vg, respectively (Figure S1B). Of the four residues in honey bee Vg, only the E residue is known to coordinate with Zn^2+^. The low conservation of E427 and the lack of other typically Zn^2+^‐coordinating residues (H/C/D/S) at both sites suggest that these locations do not take part in zinc‐binding in the honey bee.

Sullivan and Yilmaz (2018) suggest that the phosvitin domain in zebrafish Vg has a Zn^2+^‐coordinating role. Honey bee Vg does not have this domain, but the polyserine linker in the N‐terminal domain is a similar, serine‐rich region. We did not identify any C, H, D or E residues here that could support zinc‐coordination, but the linker contains 14 S residues. One or two S residues can participate in zinc‐coordination. However, this location is an unlikely candidate as it would be unprecedented to find Zn^2+^‐coordination by serines in a disordered (loop) region (Baglivo et al., 2009).

#### Assessing potential zinc coordinating residues based on protein structure modelling

##### Potential Zn ^2+^ sites in the β‐barrel subdomain of the N‐terminal region

The N‐terminal β‐barrel subdomain contains a total of seven H residues (H20, H47, H113, H193, H210, H229 and H265) and two C residues (C178 and C222) (Figure 2b). Among these, H229 is not folded in the β‐barrel subdomain (see cluster αh.1 in Figure 2c). The C residues are conserved in most of the Vg sequences included in the MSA (Figures S1A and S3A). H20 and H113 are conserved in the Vg sequences in the MSA from insect species, while the remaining H residues are less conserved. The first cluster identified in the β‐barrel subdomain, βb.1, contains H20 and H113 located in separate loop regions (Figure 2b). H265 is situated at the beginning of a β‐strand close to βb.1. In addition, we found two conserved residues (D143 and E147) in a loop region and included them in cluster βb.1 (Table 1).

The second cluster in the β‐barrel subdomain, βb.2, contains C178 and C222. The C residues form a disulfide bridge. We identified five conserved residues (E171, D172, S173, E179 and D223) close to the disulfide bridge and included them in cluster βb.2. All residues are in two neighbouring β‐strands, apart from C222 and D223 at the end of a loop region (Table 1, Figure 2b).

A short SRSSTSR sequence (residues 250–256) in the β‐barrel subdomain is proposed to bind DNA (Salmela et al., 2022). The residues are located at a β‐strand close to cluster βb.1 and βb.2 (Figures S2 and S3A).

##### Potential Zn ^2+^ sites in α‐helical subdomain and lipid binding site

We identified four clusters of conserved H/C residues (Table 1), two in the α‐helical subdomain (αh.1 and αh.2) and two in the DUF1943 (Duf.1 and Duf.2) (Figures S2C and S1B). The first cluster in the α‐helical subdomain, αh.1, contains two highly conserved H residues (H587 and H593) and two less conserved H residues (H577 and H602). All H residues are in a well‐conserved insect‐specific loop in the α‐helical subdomain (Figure S3B). The cluster also includes the well‐conserved H229. The residue is part of a loop extending from the β‐barrel subdomain, which positions H229 close to H587 and H593. The second cluster in the α‐helical subdomain, αh.2, is located at the beginning of the well‐conserved 15th α‐helix in the subdomain (Figure S3B) containing H697, C701 and E698 (Figure 2c). The cavity‐facing side of the α‐helical subdomain is close to a β‐sheet region of DUF1943. More specifically, the α‐helices are near two loops connecting the β‐strands on DUF1943. We identified three well‐conserved residues in those loops (S775, S800 and D802) and included them in cluster αh.1.

The first cluster in the DUF1943, Duf.1, contains three conserved H residues (H988, H990 and H1045). They are packed together in two loop regions at the end of two adjacent β‐strands (Figure 2c). Two conserved residues (E987 and D1046) were identified in the same loops and included in Duf.1. The second cluster in DUF194, Duf.2, contains two conserved H residues (H1000 and H1035) positioned on the same β‐sheet as Duf.1 (Figures S1B and S3C). We also found two conserved residues (D996 and S1037) in the β‐sheet region and two residues in the α‐helical subdomain (H445 and E449 in the second α‐helix of the subdomain) and included those in Duf.2.

##### Potential Zn ^2+^ sites in lipid binding site β‐sheet and the C‐terminal region

Following the DUF1943, we identified four conserved C residues (C1242, C1279, C1310 and C1324) in a β‐sheet in the lipid binding site (Figure S1C). C1242 and C1279 are highly conserved and create a disulfide bridge in the β‐sheet region. C1310 and C1324 also create a disulfide bridge connecting two α‐helices. Neither of the disulfide bridges has any conserved D, E or S residues in proximity, so the bridges are not identified as potential zinc clusters here. After this β‐sheet in the lipid binding site, the following domain is the vWF domain, which does not coordinate Zn^2+^ (Leipart et al., 2022).

The final region in honey bee Vg is the C‐terminal (residue 1635–1770). This region contains seven C and two H residues that are conserved in most Vg sequences in the MSA (Figures S1D and S3D). Six C residues create three separate disulfide bridges (Figure 2c). Two of these bridges cross each other (C1687, C1711, C1715 and C1768) and were identified as cluster Ct (Table 1). The remaining disulfide bridge and conserved C and two H residues were not nearby and therefore not considered a potential zinc‐binding site.

#### Assessing functional roles of specific zinc coordinating residues in the β‐barrel subdomain

The functional roles of zinc‐binding sites are not well‐defined for any Vg. However, a possible exception is made by the short SRSSTSR sequence close to clusters βb.1 and βb.2 in the β‐barrel subdomain that may bind DNA in honey bees (Figures 2b and S2). A recent study presents a proposed DNA‐binding motif that the subdomain recognizes (see fig. 5 in Salmela et al., 2022). Motif A is similar to the transcription factor CTCF motifs in *Drosophila melanogaster* (see Figures S5A and S5B for motif comparison). CTCF contains 11 C2H2 zinc finger factor motifs (Maksimenko et al., 2021) that bind one Zn^2+^‐ion each, shown to stabilize the structural fold in CTCF required to bind DNA (Maksimenko et al., 2021). We aligned the C2H2 motifs to the Vg sequences in our MSA (Figure S5C) and found five predicted zinc‐binding residues in the β‐barrel subdomain aligned to C and H residues in the C2H2 motifs. D143 and E147 from βb.1 align to H and D residues in the C2H2 motifs, and C178 and C222 from βb.2 align to C residues in the C2H2 motifs. In addition, H229 from αh.1 aligns to H residues in the C2H2 motifs. This configuration of zinc‐binding residues in honey bee Vg supports a functional role of the β‐barrel in DNA binding and, more specifically, suggests that Vg needs at least one Zn^2+^‐ion to stabilize the binding to DNA. We attempted to detect zinc in the β‐barrel subdomain using recombinant expression of the subdomain, but the numerous approaches did not provide a clear answer (See Supplementary Material for methodology and results).

## DISCUSSION

Our study validates that honey bee Vg binds Zn^2+^, which is suggested previously to be the most important zinc‐carrying protein in honey bee worker hemolymph (Amdam et al., 2004). The Zn:Vg ratio calculated by us, 3:1, is higher than the 1 or possible 2 zinc ions reported for each monomer of lamprey Vg (Anderson et al., 1998), which was a calculation based on measurements from the American clawed frog Vg (Auld et al., 1996; Montorzi et al., 1994). After the initial validation, we performed a sequence and structural analysis to understand the structural basis and possible functional outcomes of the zinc‐binding capacity of honey bee Vg. We identified seven zinc clusters located at different subdomains and domains.

We found two clusters in the β‐barrel subdomain. The β‐barrel subdomain is proposed to function as a transcription factor (Salmela et al., 2022). A classical feature in the DNA binding domain of transcription factor is the coordination of Zn^2+^, called zinc finger domains (Cassandri et al., 2017). The Zn^2+^‐binding assists the protein in folding, creating the structural form that can recognize DNA (Chang et al., 2010). We show a similarity between a known zinc finger protein and honey bee Vg in the DNA binding motif. The CTCF transcription factor requires coordination of Zn^2+^ at the C2H2 motifs to adopt the correct fold to bind DNA (Maksimenko et al., 2021). Similar to CTCF, the β‐barrel subdomain might also bind zinc, and through this process build a different fold than seen in our model. The MSA (Figure S5C) shows two C residues in cluster βb.2 and H229 in the β‐barrel subdomain, aligned to C and H zinc‐binding residues in CTCF. The three residues are at distant positions in the subdomain (Figure S2). The β‐barrel subdomain is presumably cleaved from honey bee Vg when translocating to the nucleus (Salmela et al., 2022). Proteolytic cleavage makes H229 available (no longer associated in the αh.1 cluster). The loop region of H229 allows for flexibility and possible association with the two C residues in cluster βb.2, creating a C2H1 site. Cluster βb.1 consists of three H residues in loop regions, which could fold similarly, resulting in a C2H2 zinc site. Taken together, we demonstrated that the β‐barrel subdomain can potentially create a C2H2 zinc site if flexibility in the loop regions is allowed for. Zinc‐binding‐related flexibility is documented for several C2H2 zinc‐binding factors, in addition to CTCF, in insects (Jauch et al., 2003; Maksimenko et al., 2021; Stubbs et al., 2011). Such flexibility could be interrupted by the bound SUMO‐tag in our expression system. The soluble yield of the β‐barrel subdomain expression system was six times lower compared to the free SUMO‐tag, suggesting a suboptimal environment for successful folding.

Cluster αh.1 is in a histidine‐rich loop in the α‐helical subdomain, a loop identified earlier as flexible and insect‐specific in honey bee Vg (Havukainen et al., 2013). A structural zinc‐binding site could increase the stability of the α‐helical subdomain by structuring the loop and stabilizing the N‐terminal domain through interaction with H229 (Figure 2c). However, proteolytic cleavage would disengage H229 from the site. We suggest that the remaining four H located in the loop (Figure 2c) could create a new structural zinc site in such situations, similar to some zinc transporter proteins (Fukada & Kambe, 2011; Tanaka et al., 2013; Zhang et al., 2019). We suggest that a conformational change induced by zinc is feasible for the flexible loop and could stabilize the α‐helical subdomain and, in turn, the lipid binding site. Cluster αh.1 is at the surface of honey bee Vg. We propose that the αh.1 cluster could sense the cellular environment more efficiently than the clusters inside the lipid cavity. Zinc transporters with similar histidine‐loop coordination sites regulate zinc homeostasis (Fukada & Kambe, 2011), supporting our findings.

Electrostatic and hydrophobic interactions between the α‐helices in the α‐helical subdomain and two β‐sheets in the DUF1943 stabilize the Vg lipid cavity (Babin et al., 1999; Biterova et al., 2019; Smolenaars et al., 2007). We suggest that structural zinc‐binding sites in the cavity could support structural stability for lipid uptake and delivery (Smolenaars et al., 2007). Clusters αh.2, Duf.1 and Duf.2 have residues at the α‐helical subdomain and the DUF1943. The clusters are in conserved (Figures S3B and S3C) and hydrophobic regions (see Figure S4 for a structural overview of the hydrophobic areas), and Duf.1 and Duf.2 consist of three H residues, typical at a structural zinc site (Dudev & Lim, 2003). Cluster αh.2 has only one H residue but has an additional C residue, a rare arrangement for a structural site, but identified in a ubiquitin‐binding protein (Lim et al., 2019; PDB‐ID: 6H3A), indicating that just two H/C residues could coordinate zinc in cluster αh.2. The structural zinc‐binding sites in zinc transporters can have an additional D residue (Fukada & Kambe, 2011), which suggests a possibility for the structural coordination event in cluster αh.2 to include a D residue (D802).

We speculated whether an interaction between H926 at the DUF1943 and the two C residues in cluster βb.2 (Figure S2) is possible. However, the residues are too distant (~30 Å) for interaction (normally ~2.0–2.4 Å; Dudev & Lim, 2003) in our model. The generally rigid β‐sheets (Perczel et al., 2005) at both positions make a conformational change induced by zinc unlikely. H926 and Duf.1 are at the same β‐sheet. However, a similar rigidness would also make interaction unlikely. Therefore, H926 is less likely to coordinate zinc, while cluster αh.2 and Duf.1, Duf.2 are feasible structural zinc‐binding sites in the lipid cavity and can coordinate zinc (eg, during transport). We suggest an optimal solution for honey bee Vg would be to carry lipid molecules and zinc in the same location so it could be released together upon delivery.

The well‐conserved disulfide bridge on the C‐terminal region presumably contributes to a stable structural fold. Such bridges are usually stable oxidative conditions (Sevier & Kaiser, 2002). Two of the disulfide bridges create an interesting arrangement and suggest the possibility for a ZnC4 coordination site, which would probably maintain the stable fold when Vg is experiencing reducing conditions. ZnC4 is a typical coordination site for redox switches (Ilbert et al., 2006; Pace & Weerapana, 2014). Redox switches can sense oxidative stress, which could generate a response of the protein to change cellular location or release zinc (Ilbert et al., 2006). We propose a similar process that can subside at the Ct cluster in honey bee Vg. This idea has some support in the observation that Vg levels in worker honey bees are positively correlated with oxidative stress resilience (Seehuus et al., 2006). It is possible that the zinc released from Vg can explain this phenomenon via binding to protective enzymes or cell membranes (Marreiro et al., 2017; Seehuus et al., 2006).

Regarding the caveats of this study, we assert that in silico analysis of the β‐barrel subdomain is not fully reflected in our experimental results, despite using several methodologies to approach this problem. Proteolytic cleavage of the SUMO‐tag resulted in low yields, and separation during purification of the tag‐free subdomain from the SUMO‐tag did not work, despite optimization. Changing the solubility‐tag to maltose‐binding protein improved expression yields slightly. However, we faced the same challenges as earlier during purification. Adding two affinity column purification steps followed by a size exclusion chromatography did not successfully separate the SUMO‐tag from the subdomain. The tagged subdomain, therefore, became the best option for element analysis. Another drawback of in silico prediction is that the orientation for some side chains can be imprecise, even when located in a confident backbone fold (Jumper et al., 2021). The residues in cluster αh.2, H593, H229 and H587 look to have an optimal side chain arrangement to coordinate zinc, creating a small triangle (Figure 2c). However, the side chains might not always be in such an optimal arrangement as seen, for example, in Duf.1. Specifically, the side chain orientation can be inaccurately predicted or could adopt another side chain orientation when zinc is present (Kluska et al., 2018). Due to these caveats, we identified the residues as potential clusters. We also assumed this for cluster βb.1, βb.2, αh.2 and Duf.2 (Figure 2b,c).

Our analysis relied on our recently published full‐length protein structure that was predicted using AlphaFold (Leipart et al., 2022). AlphaFold calculates a confidence score that evaluates the predicted structure's stereochemical integrity (Jumper et al., 2021; Mariani et al., 2013). The honey bee Vg prediction has a confidently folded backbone, with the exception of a few short loops and regions that make up approximately 13% of the Vg residues (see fig. 2 in Leipart et al., 2022). The zinc clusters that we identified here are fully embedded in confidently folded regions. However, loop regions are flexible structures (Barozet et al., 2021) and residues located in such flexible regions could potentially change the backbone fold when zinc is present, as seen in zinc regulators and zinc transporter proteins (de Angelis et al., 2010; Liu et al., 2021; Tanaka et al., 2013). We assume this could be true in our model, and this insight was applied in clusters βb.1, αh.1, αh.2 and Duf.1 (Figure 2b,c).

While the localization of one or more zinc‐binding sites to the recombinantly expressed β‐barrel remains uncertain, the presence of, on average, 3 Zn^2+^ cations per honey bee Vg molecule was determined with confidence using ICP‐MS (Figure 1). Our structural analysis illustrates where Vg can bind zinc ions, presumably one at each position. The seven sites might provide unknown flexibilities based on Vg having Zn^2+^‐ions bound at different combinations of sites depending on the protein's situation.

## EXPERIMENTAL PROCEDURES

### Collection and purification of honey bee Vg

To obtain Vg, we collected 1–10 μl honey bee hemolymph in a 1:10 dilution in 0.5 M Tris HCl (pH 7.6), using BD needles (30G), as described earlier (Aase et al., 2005). The dilution was filtered using an 0.2 μm syringe filter (Filtropur S, PES membrane). Vg was purified from honey bee hemolymph with ion‐exchange chromatography using a HiTrap Q FF 1 ml column. The sample buffer (0.5 M Tris HCl, pH 7.6) and elution buffer (0.5 M Tris HCl with 0.45 M NaCl, pH 7.6) was prepared with ion‐free water and acid‐treated (10% HNO_3_ overnight) plastic bottles to eliminate zinc contamination. Then 400–450 μl diluted hemolymph was manually injected and Vg eluted at the conductivity of 15–22 mS/cm. This specific conductivity is confirmed by earlier studies (Havukainen et al., 2011, see Figure S1 for the chromatographic fractioning of honey bee Vg; Salmela et al., 2015). All fractions from the peak were collected, pooled and concentrated using an Amicon® Ultracel 100 kDa membrane centrifuge filter. We verified the fraction purity by running SDS‐PAGE, which contained one band only of the correct size (~180 kDa). The purification protocol was repeated to produce five samples with concentrations between 1.2 and 2.8 μg/μl in 65 μl. Five blank samples were created using the same protocol; here, only sample buffer was injected. The protein concentration, measured with Qubit, confirmed that the samples contained no protein. All samples were collected in 1.5 ml Eppendorf tubes pretreated with 10% HNO_3_ for 24 h and dried at 65°C.

### Identification of zinc in honey bee Vg using ICP‐MS

ICP‐MS was performed to detect metal ions associated with purified Vg. For the ICP‐QQQ‐MS of full‐length Vg extracted from bees, 32 μl of concentrated ultra‐pure(up) nitric acid (distilled and made in‐house) was added to the 65 μl samples (purified Vg and blanks as described above). The samples were placed in a heating cabinet at 90°C for 3 h to denature the proteins and subsequently put into an ultrasound bath for 60 s to dissolve any remaining particles before analysis. Then the samples were diluted to 325 μl by adding a solution of 1% (V/V) HNO_3_ and 28.5 μg/L Rhodium (Rh, Inorganic Ventures, Christiansburg, VA) a five‐fold dilution. This gives a Rh concentration of 20 μg/L in the final sample. Rh is used as an internal standard for Zn (Gaines, 2022). For ICP‐QQQ‐MS analysis on the recombinantly expressed and purified β‐barrel, 30 μl of concentrated ultra‐pure(up) nitric acid was added to 60 μl samples. The same conditions for heat and ultrasonic bath as described above were applied. Then the samples were diluted to 300 μl by adding a solution of 1% (V/V) HNO_3_ and 30 μg/L Rh for a five‐fold dilution. This gives a Rh concentration of 21 μg/L in the final sample.

Both sample series were analysed using an Agilent Technologies 8800 ICP‐QQQ‐MS (Table S1 for instrumental parameters). The ICP‐MS was fitted with a micro nebulizer with a flow of 50 μl/min to accommodate small sample volumes. The sample introduction was a high throughput setup. External standards containing Zn (Inorganic Ventures) were used to calibrate the ICP‐MS. Rh was added to the standards in the same concentration as the samples. Zn was analysed in ammonia mode to remove any interferences with Zn, which was measured at 64 and 66 amu. Mass 64 has an isobaric overlap with ^64^Nickel (Ni); therefore, an inter‐element correction for ^64^Ni interference on ^64^Zn was performed. The internal standard Rh was measured at 103 amu.

The method limit of detection and limit of quantification was calculated as three times the SD of the buffer blank samples and 10 times the SD of the buffer blank samples. The results from the ICP‐MS was reported in μg/L and multiplied by 5 since the samples were diluted. The resulting zinc concentrations are presented in Figure 1a and Appendix_RatioCalculations.xlsx.

### Multiple sequence alignment

The sequences were aligned using Clustal Omega (McWilliam et al., 2013). The protein sequences used for the MSA were (UniProt ID): *A. mellifera* (Q868N5), *Athalia rosae* (Q17083), *Pimpla nipponica* (O17428), *Pteromalus puparum* (B2BD67), *Encarsia formosa* (Q698K6), *Bombus ignites* (B9VUV6), *Bombus hypocrite* (C7F9J8), *Solenopsis invicta* (Q7Z1M0 and Q2VQM6), *Riptortus clavatus* (O02024), *Anthonomus grandis* (Q05808), *Lethocerus deyrollei* (B1B5Z4), *Aedes aegypti* (Q16927), *Nilaparvata lugens* (A7BK94), *Graptopsaltria nigrofuscata* (Q9U5F1), *Antheraea pernyi* (Q9GUX5), *Saturnia japonica* (Q59IU3), *Periplaneta Americana* (Q9U8M0), *Blattella germanica* (O76823), *Rhyparobia maderae* (Q5TLA5), *Homalodisca vitripennis* (Q0ZUC7), *I. unicuspis* (Q91062), *Acipenser transmontanus* (Q90243), *Oreochromis aureus* (Q9YGK0), *Oncorhynchus mykiss* (Q92093), *Fundulus heteroclitus* (Q90508), *X. laevis* (P18709), *G. gallus* (P87498), *Homo sapiens* (P55157) and *Anolis carolinensis* (Q9PUB1), based on Havukainen et al. (2011). The protein sequences used in the MSA with the CTCF protein were the same as listed above, but only including the β‐barrel subdomain (residue 1 to 326 in honey bee Vg). The CTCF protein sequences used were: *D. melanogaster* (Q9VS55), *Anopheles gambiae* (Q4G266), *Bombyx mori* (H9IXV8), *Tribolium castaneum* (D6WGY1), *A. mellifera* (A0A7M7MWC7 and A0A7M7MWF4) and *Nasonia vitripennis* (A0A7M7H6S9). The CTCF proteins were identified through BLAST (Altschul et al., 1990), and the species represent the evolutionary relationship between *A. mellifera* and *D. melanogaster* (Honeybee Genome Sequencing, 2006). See Figure S5C for MSA.

### Identification of clusters

We used the full‐length AlphaFold prediction of honey bee Vg for our structural analysis (Leipart et al., 2022). The first step was to identify conserved H and C residues (see Figure S1 for MSA). Second, we inspected their positions in 3D space to determine the distance and positions in relation to each other. Zinc usually coordinates with a minimum of four residues, so if our initial search did not identify at least four H/C in proximity, we went back to the MSA to identify adjacent (in sequence or 3D space) conserved S/D/E residues or regions of conserved residues (potential coordination through backbone association). Since the structural model lacks water molecules, we could not account for possible coordination involving hydrogen bonds from water molecules. The potential zinc‐binding sites found in this way are called clusters. The MSA was uploaded to ConSurf (Ashkenazy et al., 2016) to create a PyMol script colouring atoms based on the degree of conservation. The results are presented in Figure S3. Finally, we evaluated the hydrophobic contrast of each cluster using Dudev and Lim's (2003) formalism and using the PyMol command based on the Eisenberg hydrophobicity scale (Eisenberg et al., 1984). The results are presented in Figure S4. The *A. mellifera* (Q868N5) sequence was input to ZincBind (Ireland & Martin, 2019), MotifScan (Pagni et al., 2007) and MOTIF Search (GenomeNet, Kyoto University Bioinformatics Center). Only MotifScan resulted in a positive hit.

### Limited proteolysis

A limited proteolysis analysis was performed to obtain the 40 kDa fragment of the β‐barrel subdomain. Purified Vg was dephosphorylated with Lambda Protein Phosphatase (New England BioLabs, Ipswich, MA). The samples were incubated with 1 μl Lambda Protein Phosphatase, 5 μl 10× NEBuffer for Protein MetalloPhosphatases and 5 μl of 10 mM MnCl_2_, for 30 min at 30°C. Then 6.5 ng of dephosphorylated Vg was digested with 5 and 10 units of caspase‐1 (Sigma–Aldrich, Saint‐Louis, MO) for 2 h at 37°C, and with 0.01, 0.1 and 1 unit of chymotrypsin (Sigma–Aldrich) for 30 min at 25°C. Two standards (ThermoFisher PageRuler™ unstained and pre‐stained High Range Protein Ladder), unphosphorylated and undigested full‐length Vg, and the digested samples were run on 4%–20% SDS‐PAGE gel (Bio‐Rad, Berkeley, CA) under reducing conditions.

### Recombinant protein and preparation for element analysis

The β‐barrel (amino acid 21–323) protein subdomain was produced by Genscript Biotech, as described in the following. The gene coding for the polypeptide was subcloned by Genscript into a pET30a(+) vector, with an N‐terminal His tag and a SUMO solubility tag. The construct was expressed in *Escherichia coli* Arctic Express (D3), and a single colony was inoculated into an LB medium containing kanamycin, and also including 100 μM Zn^2+^. The culture was incubated in 37°C at 200 rpm, until a cell density of at least optical density (OD) = 0.6 at 600 nm was reached. The culture was then induced by adding 0.5 mM IPTG and grown at 15°C for 16 h. The target protein was resolubilized and purified using Ni‐affinity purification. The product of this Genscript service, that is, the SUMO tagged β‐barrel subdomain, was used in ICP‐MS analysis.

To control for the presence of Zn^2+^ in the buffer and any ability of SUMO to coordinate Zn^2+^, we made three sets of blank samples: buffer blank and two set of samples with SUMO (human, His‐tag, Enzo Life Sciences, Farmingdale, NY). One set of SUMO samples was incubated with 25 μM ZnCl_2_ at 4°C for 1 h, the other was not. The buffers in all four sample sets (SUMO tagged β‐barrel, buffer only, SUMO incubate with Zn^2+^ and SUMO with no Zn^2+^ incubation) were then exchanged to 0.5 M Tris HCl with 0.225 M NaCl in preparation for ICP‐MS.

The expression system was also used to produce samples for UV–Vis spectroscopy, NMR spectroscopy and intrinsic tryptophan fluorescence spectroscopy (see Supplementary.docx and Figure S8). For this the LB medium used for expression included 42 μM Zn^2+^ (in addition to the 8 μM present in the unmodified medium, for a total of 50 μM) or 50 μM Co^2+^, and one included both 42 μM Zn^2+^ and 50 μM Co^2+^. The SUMO tag only expression system with the same three conditions as above was also ordered to control for false positive results related to SUMO metal binding.

## AUTHOR CONTRIBUTIONS

**Vilde Leipart:** Conceptualization (lead); data curation (equal); formal analysis (equal); investigation (equal); methodology (equal); project administration (lead); validation (equal); visualization (lead); writing – original draft (lead); writing – review and editing (equal). **Øyvind Enger:** Formal analysis (equal); investigation (equal); methodology (equal); validation (equal); writing – original draft (supporting); writing – review and editing (equal). **Diana Cornelia Turcu:** Formal analysis (equal); investigation (equal); methodology (equal); writing – review and editing (equal). **Olena Dobrovolska:** Investigation (supporting); methodology (supporting); writing – review and editing (equal). **Finn Drabløs:** Investigation (supporting); methodology (supporting); visualization (supporting); writing – review and editing (equal). **Øyvind Halskau:** Conceptualization (supporting); data curation (equal); formal analysis (equal); investigation (equal); methodology (equal); project administration (supporting); supervision (supporting); validation (equal); visualization (supporting); writing – original draft (supporting); writing – review and editing (lead). **Gro V. Amdam:** Conceptualization (supporting); formal analysis (supporting); funding acquisition (lead); methodology (supporting); project administration (supporting); resources (lead); supervision (lead); validation (supporting); writing – review and editing (lead).

## CONFLICT OF INTEREST

The authors declare no conflict of interest.

## Supporting information


**Figure S1.** Multiple sequence alignment. Snapshots of the multiple sequence alignment. All the included species are noted with their UniProt ID. Residues included in clusters are in bold colours (αh.1: green, αh.2: red, Duf.1: dark blue, Duf.2: orange, Ct: yellow, βb.1: pink and βb.2: cyan). Some alignment regions are excluded (noted with ‘…’) since they are not relevant to this study or have significant gaps. (A) The residues from βb.1, βb.2 and one residue from cluster αh.1 are in the β‐barrel subdomain. In addition, the DNA binding motif (pink box) is part of this subdomain. The conserved residues are coloured (bold black). (B) Cluster αh.1, αh.2, Duf.1 and Duf.2 are in the lipid binding site (DUF1943). In addition, the suggested zinc coordinating residues from studies in Lamprey (grey bold) and the MotifScan zinc‐binding site (yellow box) are found in this region. H926 is coloured (black bold). (C) No cluster was identified in the region before the vWF domain, but the conserved disulfide bridges residues are coloured (brown bold). (D) Cluster Ct is in the C‐terminal region, where all the conserved C and H residues are marked.
**Figure S2.** MotifScan. The zinc‐binding motif (yellow) identified by MotifScan is located in the DUF1943 domain (purple) but extends into the cavity of the β‐barrel (green). The predicted zinc‐binding H residue (H926) is shown as a stick. Cluster Duf.1 (blue spheres), βb.1 (magenta spheres), βb.2 (cyan spheres), H229 from αh.1 (green stick) and the DNA binding motif (pink) is in close proximation to this predicted zinc‐binding motif.
**Figure S3.** Conservation. Residues are coloured using ConSurf, based on the MSA. The low to highly conserved residues are coloured from light blue to dark pink (scale presented in the lower‐right corner). Both the secondary structures and the spheres (representing the clusters) are coloured according to this scale. (A) The buried residues in the β‐barrel subdomain are well 6 conserved, including the residues in cluster βb.1, βb.2 and the DNA binding motif β‐sheet. The regions closer to the surface are less conserved. (B) The α‐helices in the α‐helical subdomain are well‐conserved. The residues in Cluster αh.1 and αh.2 are also conserved. (C) One of the β‐sheet in the DUF1943 domain includes cluster Duf.1, Duf.2 and the zinc motif identified by MotifScan. The conservation of the residues in the β‐sheet are variable, but the clusters and zinc motif are conserved. As shown in the MSA, residue H926 is not conserved. (D) The C‐terminal is generally not conserved, except the four residues presented as cluster Ct and the third disulfide bridge (labelled).
**Figure S4.** Hydrophobicity. The surface and secondary structure are coloured using the Eisenberg hydrophobicity scale (scale presented in the lower‐left corner). In both panels, the clusters are shown as spheres and marked with a blue dotted circle. Their respective domains are presented as surface and cartoon. (A) Cluster αh.2 is the position between the highly hydrophobic core of the lipid binding site (β‐sheet) and the polar surface of the α‐helical subdomain. (B) Cluster Duf.1 and Duf.2 are positioned on the same β‐sheet that make up one side of the lipid binding site (highly hydrophobic), while the other side is facing the surface and is more polar.
**Figure S5.** Logo representation of DNA binding site motifs and sequence analysis of CTCF. (A) The most significant motif (motif A) found by Salmela et al. (2021) for Vg‐DNA binding sites, as shown in Figure 5. (B) The motif for CTCF in *Drosophila melanogaster* from the JASPAR database (Castro‐Mondragon et al., 2021) (matrix MA0531.1). The logo representation was made with WebLogo3 (Schneider and Stephens, 1990, Crooks et al., 2004). (C) Residue 140 to 233 in the β‐barrel subdomain, using the same species as in the full‐length MSA (Figure S1), aligned to CTCF proteins. The conserved residues from the β‐barrel subdomain, identified in the CTCF proteins, are in bold.
**Figure S6.** Proteolysis of honey bee Vg by caspase‐1 and chymotrypsin. The black arrow emphasizes the probable 40 kDa cleavage products of the full‐length Vg (flVg) with 5 and 10 units of caspase‐1 in lanes 3 and 4, respectively. We did not identify a clear 150 kDa band. The smaller bands outside the range of the standard could be the lambda protein phosphates (25 kDa) or caspase‐1 (30 kDa). The chymotrypsin (lane 5 to 7) cleaves Vg completely into small fragments, and no 40 kDa band was identified. The smaller bands outside the range of the standard could be lambda protein phosphates (25 kDa) or chymotrypsin (25 kDa).
**Figure S7.** ICP‐MS results for the β‐barrel subdomain. The zinc concentration was measured with ICP‐MS for the x5 samples of SUMO tagged β‐barrel subdomain (bb), sample buffer (blk), non‐incubated SUMO tag (Sblk) and SUMO‐tag incubated with Zn2+ (SZnblk). The mean and the SD of the mean are indicated for each group.
**Figure S8.** Spectroscopic analyses of SUMO‐fusion proteins expressed in Zn2+, Zn2+ and Co2+, and Co2+ medium. (A) UV–Vis spectra of protein expressed in medium enriched with Zn2+ (green traces), Zn2+ and Co2+ (blue traces), and Co2+ (red traces). Expressions of the Sumo tag only are represented by dashed lines, and the SUMO beta‐barrel fusion protein is represented by whole lines. (B) Amide region from 1H NMR spectra of SUMO beta‐barrel fusion protein expressed in medium enriched with Zn2+ (green trace), Zn2+ and Co2+ (blue trace), and Co2+ (red trace). (C) Intrinsic tryptophan fluorescence spectra of SUMO beta‐barrel fusion protein expressed in medium enriched with Zn2+ (green trace), Zn2+ and Co2+ (blue traces) and Co2+ (red trace).
**Table S1.** Instrumental parameters used for Agilent 8800 ICP‐MS.

## Data Availability

All data presented here are included in the main article or the supplementary material (Supplementary.docx and Appendix_RatioCalculations.xlsx). The structural data for honey bee Vg was published earlier and supplemented there (Leipart et al., 2022).
