# Supplementary material for: Resolving the zinc binding capacity of honey bee vitellogenin and locating its putative binding sites
Source: Insect Mol Biol. 2022 Aug 23;31(6):810–20. doi: 10.1111/imb.12807 (PMC9804912; doi:10.1111/imb.12807)
Supplement: Supplementary file 1 — Figure S1. Multiple sequence alignment. Snapshots of the multiple sequence alignment. All the included species are noted with their UniProt ID. Residues included in clusters are in bold colours (αh.1: green, αh.2: red, Duf.1: dark blue, Duf.2: orange, Ct: yellow, βb.1: pink and βb.2: cyan). Some alignment regions are excluded (noted with ‘…’) since they are not relevant to this study or have significant gaps. (A) The residues from βb.1, βb.2 and one residue from cluster αh.1 are in the β‐barrel subdomain. In addition, the DNA binding motif (pink box) is part of this subdomain. The conserved residues are coloured (bold black). (B) Cluster αh.1, αh.2, Duf.1 and Duf.2 are in the lipid binding site (DUF1943). In addition, the suggested zinc coordinating residues from studies in Lamprey (grey bold) and the MotifScan zinc‐binding site (yellow box) are found in this region. H926 is coloured (black bold). (C) No cluster was identified in the region before the vWF domain, but the conserved disulfide bridges residues are coloured (brown bold). (D) Cluster Ct is in the C‐terminal region, where all the conserved C and H residues are marked. Figure S2. MotifScan. The zinc‐binding motif (yellow) identified by MotifScan is located in the DUF1943 domain (purple) but extends into the cavity of the β‐barrel (green). The predicted zinc‐binding H residue (H926) is shown as a stick. Cluster Duf.1 (blue spheres), βb.1 (magenta spheres), βb.2 (cyan spheres), H229 from αh.1 (green stick) and the DNA binding motif (pink) is in close proximation to this predicted zinc‐binding motif. Figure S3. Conservation. Residues are coloured using ConSurf, based on the MSA. The low to highly conserved residues are coloured from light blue to dark pink (scale presented in the lower‐right corner). Both the secondary structures and the spheres (representing the clusters) are coloured according to this scale. (A) The buried residues in the β‐barrel subdomain are well 6 conserved, including the residues in cl [file IMB-31-810-s001.pdf]

Supplementary Material

Supplementary methods for detection of zinc in the β-barrel subdomain

To begin validating the role of zinc in the honey bee Vg β-barrel subdomain, we initially attempted to obtain a sample through dephosphorylation and proteolysis of native Vg at the polyserine linker. However, this was unsuccessful. A weak 40kDa band was obtained under certain conditions, but we could not validate its identity (see Figure S6 for SDS-PAGE gel). Therefore, the subdomain was expressed in *E. coli* with a solubility tag (SUMO) by Genscript Biotech, and ICP-MS was repeated. The Zn^{2+} concentration for the tagged β-barrel subdomain samples was significantly higher than the negative controls of buffer samples (Mann-Whitney U test: w = 0, p-value = 0.0106) (see Figure S7 for ICP-MS results), but not significantly different from samples of the free SUMO-tag, including all samples (Mann-Whitney U test: w = 4, p-value = 0.0937). Excluding one outlier in the SUMO-tag sample set, however, yielded significant results (Mann-Whitney U test: w = 0, p-value = 0.0195). This outcome seems encouraging, but the tagged β-barrel subdomain was exposed to Zn^{2+} during expression in culture, while the free SUMO-tag was synthetically produced without similar opportunity for zinc-binding. We attempted to develop systems to control for this confounding factor (see supplementary.docx and Figure S7 and S8), but without sufficient success.

ICP-MS continued

We attempted to control for Zn^{2+} interacting with the SUMO-tag by incubating the SUMO-tag with Zn^{2+} before ICP-MS. Incubation resulted in significantly higher Zn^{2+} levels in SUMO-tag samples compared to the non-incubated tagged β-barrel subdomain (Mann-Whitney U test: w = 0, p-value = 0.0114). The difference in concentration indicated that the incubation caused the association of Zn^{2+} with the SUMO-tag. However, we could not rule out more specific Zn^{2+}-binding to SUMO. The net determined Zn^{2+} concentration for the β-barrel subdomain was much lower compared to the concentration for native full-length Vg. Figure 1B shows a mean of 1524.0000 µg/L (SD ± 261.6562), while Figure S7 shows the mean as 64.2000 µg/L (SD ± 13.4350), and the significance suggested 0.01 bound Zn^{2+} per β-barrel molecule. We attempted to examine this result with an independent approach and expressed the β-barrel subdomain in the presence of $Co^{2+,}$ attempting to displace Zn^{2+} with this cation. Co^{2+} is considered a good structural and functional model for studying Zn^{2+}-binding sites as the coordination is similar and exchange for Zn^{2+} to Co^{2+} occurs in nature (Lane and Morel, 2000, Shumilina et al., 2014). In contrast to Zn^{2+}, Co^{2+} coordination causes a readily detected change in the protein's UV-Vis spectrum (Bertini and Luchinat, 1984, Shumilina et al., 2014, Sivo et al., 2017). The conducted experiments are presented below.

UV-Vis Spectroscopy

SUMO fusion proteins were expressed and purified as described in the main manuscript (i.e., expressed in the presence of Zn^{2+} [42 µM], Zn^{2+} and Co^{2+} [42 µM and 50 µM, respectively], and Co2+ [50 µM]) to identify the presence of Zn^{2+}-binding sites by Co^{2+} substitution.

Initially, in a storage buffer consisting of PBS at pH 7.4, 10% glycerol, and 0.5 M L-arginine, they were centrifuged at 17000xg for 10 min. Supernatants were concentrated using Amicon

filters with 10 kDa cut-off at 3250xg for 30 min, producing approximately 4 mg/mL protein samples. These were transferred to the UV-VIS cuvette (path length 10 mm, 500 µL capacity, Hellma item number 108-002-10-40). A UV-Vis spectra in the range of 200–800nm at room temperature were acquired. If Co^{2+} successfully replaced Zn^{2+}, we would have expected to see a distinct Co^{2+}-specific peak pattern near the 500–750 nm range (Sivo et al., 2017, Lane and Morel, 2000). However, we did not observe this (negative results are presented in Figure S8A). The samples were then used for NMR and intrinsic tryptophan fluorescence spectroscopy (see below).

NMR Spectroscopy

Co^{2+} can create a paramagnetic shift in protein NMR spectra (Lane and Morel, 2000). Therefore, we transferred stocks of SUMO-fusion proteins exposed to Zn^{2+}, Zn^{2+} Co^{2+}, and Co^{2+} (as described above) to NMR tubes. D_2O up to 5% v/v was added. We then acquired 1D proton spectra (25°C, water suppression using Watergate, 512 scans, processed using exponential multiplication with a line broadening of 0.3 Hz), and inspected the spectra without finding any significant differences between the samples (Figure S8B).

Intrinsic Tryptophan Fluorescence Spectroscopy

We also looked for fold changes in response to divalent cation(s) present during expression. To do this, we transferred 5 µL of the NMR samples (described above) into 300 PBS buffer at pH 6.5 in a quartz cuvette (path length 5 mm) and conducted an emission scan (excitation wavelength 295 nm, slit widths 5 nm, 310–500 nm). The spectra, which primarily provide information on the microenvironment of the tryptophans in the protein (Knappskog and Haavik, 1995, Takita et al., 2003) did not show any significant difference.

Table S1. Instrumental parameters used for Agilent 8800 ICP-MS

**ICP-MS**	**Settings[1]**	**Settings[2]**
RF power	1600 W	1600 W
Plasma gas	15 L/min	15 L/min
Auxiliary gas	0,9 L/min	0,9 L/min
Nebulizer gas	0,90 L/min	0,95 L/min
Makeup gas	0,30 l/min	0,20 l/min
Nebulizer pump	0,32 rpm	0,32 rpm
Sampler/skimmer	Ni	Ni
Sample Depth	8,0 mm	8,0 mm
**Data registration for ICP-MS**		
Rinse time	20 s	20 s
Flush time	50 s	50 s
Read delay	15 s	15 s
Scanning mode	peak hop	peak hop
Points/spectral peak	1	1
Sweeps/reading	10	10
Replicates	5	5
P/A detector (puls/analog)	on	on
Temperature spray chamber	12 °C	2 °C

1) First analysis series (full-length Vg)
2) Second analysis series (β-barrel subdomain)

Figure legends

Figure S1: Multiple sequence alignment. Snapshots of the multiple sequence alignment. All the included species are noted with their UniProt ID. Residues included in clusters are in bold colors (αh.1: green, αh.2: red, Duf.1: dark blue, Duf.2: orange, Ct: yellow, βb.1: pink and βb.2: cyan). Some alignment regions are excluded (noted with "…") since they are not relevant to this study or have significant gaps. **A)** The residues from βb.1, βb.2, and one residue from cluster αh.1 are in the β-barrel subdomain. In addition, the DNA binding motif (pink box) is part of this subdomain. The conserved residues are colored (bold black). **B)** Cluster αh.1, αh.2, Duf.1 and Duf.2 are in the lipid binding site (DUF1943). In addition, the suggested zinc coordinating residues from studies in Lamprey (gray bold) and the MotifScan zinc-binding site (yellow box) are found in this region. H926 is colored (black bold). **C)** No cluster was identified in the region before the vWF domain, but the conserved disulfide bridges residues are colored (brown bold). **D)** Cluster Ct is in the C-terminal region, where all the conserved C and H residues are marked.

Figure S2: MotifScan. The zinc-binding motif (yellow) identified by MotifScan is located in the DUF1943 domain (purple) but extends into the cavity of the β-barrel (green). The predicted zinc-binding H residue (H926) is shown as a stick. Cluster Duf.1 (blue spheres), βb.1 (magenta spheres), βb.2 (cyan spheres), H229 from αh.1 (green stick), and the DNA binding motif (pink) is in close proximation to this predicted zinc-binding motif.

Figure S3: Conservation. Residues are colored using ConSurf, based on the MSA. The low to highly conserved residues are colored from light blue to dark pink (scale presented in the lower-right corner). Both the secondary structures and the spheres (representing the clusters) are colored according to this scale. **A)** The buried residues in the β-barrel subdomain are well

conserved, including the residues in cluster βb.1, βb.2, and the DNA binding motif β-sheet. The regions closer to the surface are less conserved. **B)** The α-helices in the α-helical subdomain are well conserved. The residues in Cluster αh.1 and αh.2 are also conserved. **C)** One of the β-sheet in the DUF1943 domain includes cluster Duf.1, Duf.2, and the zinc motif identified by MotifScan. The conservation of the residues in the β-sheet are variable, but the clusters and zinc motif are conserved. As shown in the MSA, residue H926 is not conserved. **D)** The C-terminal is generally not conserved, except the four residues presented as cluster Ct and the third disulfide bridge (labeled).

Figure S4: Hydrophobicity. The surface and secondary structure are colored using the Eisenberg hydrophobicity scale (scale presented in the lower-left corner). In both panels, the clusters are shown as spheres and marked with a blue dotted circle. Their respective domains are presented as surface and cartoon. **A)** Cluster αh.2 is the position between the highly hydrophobic core of the lipid binding site (β-sheet) and the polar surface of the α-helical subdomain. **B)** Cluster Duf.1 and Duf.2 are positioned on the same β-sheet that make up one side of the lipid binding site (highly hydrophobic), while the other side is facing the surface and is more polar.

Figure S5: Logo representation of DNA binding site motifs and sequence analysis of CTCF. A) The most significant motif (motif A) found by Salmela *et al.* (2021) for Vg-DNA binding sites, as shown in Figure 5. **B)** The motif for CTCF in Drosophila melanogaster from the JASPAR database (Castro-Mondragon et al., 2021) (matrix MA0531.1). The logo representation was made with WebLogo3 (Schneider and Stephens, 1990, Crooks et al., 2004). **C)** Residue 140 to 233 in the β-barrel subdomain, using the same species as in the full-length MSA (Figure S1),

aligned to CTCF proteins. The conserved residues from the β-barrel subdomain, identified in the CTCF proteins, are in bold.

Figure S6. Proteolysis of honey bee Vg by caspase-1 and chymotrypsin. The black arrow emphasizes the probable 40 kDa cleavage products of the full-length Vg (flVg) with 5 and 10 units of caspase-1 in lanes 3 and 4, respectively. We did not identify a clear 150 kDa band. The smaller bands outside the range of the standard could be the lambda protein phosphates (25 kDa) or caspase-1 (30 kDa). The chymotrypsin (lane 5 to 7) cleaves Vg completely into small fragments, and no 40 kDa band was identified. The smaller bands outside the range of the standard could be lambda protein phosphates (25 kDa) or chymotrypsin (25 kDa).

Figure S7. ICP-MS results for the β-barrel subdomain. The zinc concentration was measured with ICP-MS for the x5 samples of SUMO tagged β-barrel subdomain (bb), sample buffer (blk), non-incubated SUMO tag (Sblk), and SUMO-tag incubated with Zn^{2+} (SZnblk). The mean and the standard deviation of the mean are indicated for each group.

Figure S8. Spectroscopic analyses of SUMO-fusion proteins expressed in Zn^{2+}, Zn^{2+} and Co^{2+}, and Co^{2+} medium. A) UV-Vis spectra of protein expressed in medium enriched with Zn^{2+} (green traces), Zn^{2+} and Co^{2+}(blue traces), and Co^{2+} (red traces). Expressions of the Sumo tag only are represented by dashed lines, and the SUMO beta-barrel fusion protein is represented by whole lines. **B)** Amide region from ^1H NMR spectra of SUMO beta-barrel fusion protein expressed in medium enriched with Zn^{2+} (green trace), Zn^{2+} and Co^{2+}(blue trace), and Co^{2+} (red trace). **C)** Intrinsic tryptophan fluorescence spectra of SUMO beta-barrel fusion protein expressed in medium enriched with Zn^{2+} (green trace), Zn^{2+} and Co^{2+}(blue traces), and Co^{2+} (red trace).

References

BERTINI, I. & LUCHINAT, C. 1984. High spin cobalt(II) as a probe for the investigation of metalloproteins. *Adv Inorg Biochem,* 6**,** 71-111.

CASTRO-MONDRAGON, J. A., RIUDAVETS-PUIG, R., RAULUSEVICIUTE, I., BERHANU LEMMA, R., TURCHI, L., BLANC-MATHIEU, R., LUCAS, J., BODDIE, P., KHAN, A., MANOSALVA PÉREZ, N., FORNES, O., LEUNG, TIFFANY Y., AGUIRRE, A., HAMMAL, F., SCHMELTER, D., BARANASIC, D., BALLESTER, B., SANDELIN, A., LENHARD, B., VANDEPOELE, K., WASSERMAN, W. W., PARCY, F. & MATHELIER, A. 2021. JASPAR 2022: the 9th release of the open-access database of transcription factor binding profiles. *Nucleic Acids Research*.

CROOKS, G. E., HON, G., CHANDONIA, J. M. & BRENNER, S. E. 2004. WebLogo: a sequence logo generator. *Genome Res,* 14**,** 1188-90.

KNAPPSKOG, P. M. & HAAVIK, J. 1995. Tryptophan Fluorescence of Human Phenylalanine Hydroxylase Produced in Escherichia coli. *Biochemistry,* 34**,** 11790-11799.

LANE, T. W. & MOREL, F. M. 2000. Regulation of carbonic anhydrase expression by zinc, cobalt, and carbon dioxide in the marine diatom Thalassiosira weissflogii. *Plant physiology,* 123**,** 345-352.

SCHNEIDER, T. D. & STEPHENS, R. M. 1990. Sequence logos: a new way to display consensus sequences. *Nucleic Acids Res,* 18**,** 6097-100.

SHUMILINA, E., DOBROVOLSKA, O., DEL CONTE, R., HOLEN, H. W. & DIKIY, A. 2014. Competitive cobalt for zinc substitution in mammalian methionine sulfoxide reductase B1 overexpressed in E. coli: structural and functional insight. *Journal of biological inorganic chemistry : JBIC : a publication of the Society of Biological Inorganic Chemistry,* 19**,** 85-95.

SIVO, V., D'ABROSCA, G., RUSSO, L., IACOVINO, R., PEDONE, P. V., FATTORUSSO, R., ISERNIA, C. & MALGIERI, G. 2017. Co(II) Coordination in Prokaryotic Zinc Finger Domains as Revealed by UV-Vis Spectroscopy. *Bioinorg Chem Appl,* 2017**,** 1527247.

TAKITA, T., NAKAGOSHI, M., INOUYE, K. & TONOMURA, B. I. 2003. Lysyl-tRNA Synthetase from Bacillus stearothermophilus: The Trp314 Residue is Shielded in a Non-polar Environment and is Responsible for the Fluorescence Changes Observed in the Amino Acid Activation Reaction. *Journal of Molecular Biology,* 325**,** 677-695.

Figure S1

A) β-barrel MSA (residue 19 to 266)

Position markers: 20, 113, 143, 171, 178

```
A. mellifera (Q868N5)       QHNWQ...EISDQMLELRDLPISG--KPFQIRMK--HGLIRDLIVDRDVPTWEVNILKSIVGQLQVDTQGENAVKVNSVQVPTDD--EPYASFKAMEDSVGGKCEVLYDIAPLSD
A. rosae (Q17083)           QHAWK...PLPEKKTNYQKFELSE--KPFEIKFK--NGVIDDLVVEKSLPTWQVNIIKSIASQLQVDTQGENAMKSRYNQEPEGD--QAYATYKAMEDTVTGECEVLYDISPLPE
P. nipponica (O17428)       LQAWK...EIPDHKLEMKQFPMSE--KVFEIKMK--HGVVRDLIVDKDVPTWEVNVLKSIVSQLQVDTQGENLMASKYNQEPEDE--GVTAMFKTMEDTVGGRCEVLYDINPLPE
P. puparum (B2BD67)         QNGWK...EIPQNQMNMQTFPLSG--KPFEIKSK--NGVVRDLIVDKNVPTWEVNVLKSIVSQLQVDTQGENAIRSKHNQFPEGK--QPYALFKVMEDSVGGMCEVLYDVSALPD
E. formosa (Q698K6)         LEGWK...EIPRNQMNSQGLPLSN--QPFEIKIK--NGVVRDLIVNKNVPTWEVNILKAIVSQLQVDARGQNAEYSKHNQLPEGK--QPYAIFKTREDSVSGRSEVTYDISPLPE
B. ignites (B9VUV6)         DHGWH...EISDQMLELRDMPLSG--KPFNIKLK--HGVIRDIIVDRTIPTWEVNILKSIVSQLQVDSLGENAIRTSEMQIPTDE--HPYGMFRAMEDSVGGKCEVLYDITPLPE
B. hypocrite (C7F9J8)       DHGWH...EISDQMLELRDMPLSG--KPFNIKLK--HGVIRDIIVDRTIPTWEVNILKSIVSQLQVDSLGENAIRTSETQIPTDE--YPYGMFRAMEDSVGGKCEVLYDIMPLPE
S. invicta (Q7Z1M0)         DHAWE...EIAD--LKFDELSMSG--KSFEIKLK--HGVIRDVLIDQDVLTWEVNLIKSIVSQLQVDLQGENVIASSDNQIPDDS--QPFGVYKAMEDSVGGKCEVLYSITPVPE
S. invicta (Q2VQM6)         EHAWE...FDDPKNVNYKRMPMSE--KPFEIKLK--HGIIRDLLFDRDVPTWEVNMMKAIVGQLQVDTQGENAINSKSIQVPSDE--SFAATFKAMEDSVSGKCEVLYEITPLTV
R. clavatus (O02024)        DNPWK...EIQENELQYNELPISK--DPFEIHFR--NGVIEKMVVNKDIPQWQLNFYKSIASQFQVDTQGKNLKDSKINLVPSGQSGEPMGVYKTMEDSVNGIYETLYDISVLPE
A. grandis (Q05808)         SPAWK...EIPDSQISYKQLQLSQ--KPFQLVLE--NGLIKRLIVEKDTLNWEANIIKSIVSQFQMDLQGENALQNPTSSFPTNE--YMDAVFKTMEETVTGKTETIYDIHRLPE
L. deyrollei (B1B5Z4)       ATAWK...EIPDNKLHWQPMPLAG--KPFELKLK--NGTVDRMYVDKSVPTWEENWYKSIASQLQIDTAARNLQKSRINQLPAMH--KPMGVYKTMEDTVTGECETVYDVSPLPD
A. aegypti (Q16927)         FSSWM...KFYHDMFKFQPMPMSS--KPFGIRYH--KGAIKGLYVEKTIPNNEVNILKAWISQLQVDTRGANLMHSSKPIHPSKN--EWNGHYKVMEPLVTGECETHYDVNLIPA
N. lugens (A7BK94)          SGPWN...ELRSNELQYKQLPLSQANQVFQVNYK--QGAVRSLQVNRNTPTWELNMIKGFVSLFQVDVTGQNAIKSRRNIVPNGQ--QVSGSFKVMEDSVTGKCETHYDVDELPM
G. nigrofuscata (Q9U5F1)    NTGFE...QLPGD-ITMKPLYLNS--SPVEVHYR--NGVVDSIRVSKNMPTYELNIIKSIVSQLQFDTEGENIQKSRSNSLFDNS-DDPVVTFKTMEASVTGKCETLYDFSPLLK
A. pernyi (Q9GUX5)          YSPWQ...-VPE-NLKYQPVQNLD--KPFEISFD--GGRINTFNLPSSVSLQHENLLKGLISTLQLDLSTHRNIHGSQDSYDR-E--QKQGLFRKMETDVTGDCETLYSVSPVAS
S. japonica (Q59IU3)        LSPWQ...-LPY-NLNYQPVQKLD--NPFEISFE--GGRINTLSLPSSMSLPHENLLKGLISTLQLDLSTHRNIQGSQNRYDQ-E--QKQGLFRKMETDVTGDCETLYTVSPVAS
P. Americana (Q9U8M0)       LQEWD...EIEI--HDWQQLSALK--DAFAIILQ--NGHIVNVKVKDSTPNWAVNILKGILSTLQVNTQADNLVRHRYNILPNAS--TDSAVYSIRETTITGECEVEYDVSPLPA
B. germanica (O76823)       TPGWL...SVPDEKLHWDVVPMSQ--QPFQIELNS-RGEVRKLRVNKFVELWEINMIKAIISQLQVVVDE------------------DKKVYRVFESTVTGRCEALYEVDHLYP
R. maderae (Q5TLA5)         PRGWI...NIPDSQLKWKDLPLRQ--DTFDAHINDTSGEVEILYVNSQLQLWEVNVIKGLLSQIQLTTQP-----------------SFKPVYRVKESIITGRCHTLYDFSPLLK
H. vitripennis (Q0ZUC7)     NLAWK...PIPESQRHYQPIPMSN--KPFQLVYK--NGVISRMVVSKGVPTWELNILKSIASQFQVDTQAENLQKSRINSLPTQE--TVNGVYKTMEDSVSGECETLYDISPLPK
I. unicuspis (Q91062)       DAQFQ...FYPLTAGYDHFIQQLE--VPVRFDYS--AGRIGDIYAPPQVTDTAVNIVRGILNLFQLSLKK------------------NQQTFELQETGVEGICQTTYVVQEG--
A. transmontanus (Q90243)   EPSFS...FYPASKLTQALASQLT--QPIKFQYR--NGQVGDIFASEDVSDTVLNIQRGILNMLQLTIKT------------------TQNVYGLQENGIAGICEASYVIQED--
O. aureus (Q9YGK0)          APGFA...FHPATKLTTALATQLS--TPIKFEYT--NGVVGRLAAPPGVSTTVLNIYRGIINLLQLNVKK------------------TQNVYEMQESGAHGVCKTNYVIRED--
O. mykiss (Q92093)          APDFA...FVPAAKLTSALAAQFS--IPIKFEYA--NGVVGKVLAPTAVSETVLNVHRGILNILQLNIKK------------------TQNVYELQEAGAQGVCKTHYVIRED--
F. heteroclitus (Q90508)    APEFA...AVPATKLTAALAPQLA--IPIKFEYT--NGVVGKVFAPEEVSTLVLNIYRGILNILQLNIKK------------------THKVYDLQEVGTQGVCKTLYSISED--
X. laevis (P18709)          EPVFS...FTRSSKLTQALAEQLT--KPARFEYS--NGRVGDIFVADDVSDTVANIYRGILNLLQVTIKK------------------SQDVYDLQESSVGGICHTRYVIQED--
G. gallus (P87498)          QPDFG...FSRSLKLTQALTGQLS--IPIKFEYS--NGHVGNLMAPDSVSDDGLNIYRGILNILELSLKK------------------MQHSYSIQEAGIGGICNTTYAIQEN--
H. sapiens (P55157)         GLSLN...---SKIMGKENLEALQ--RPTLLHLI--HGKVKEFYSYQNEAVAIENIKRGLASLFQTQLSS------------------G----TTNEVDISGNCKVTYQAHQD--
A. carolinensis (Q9PUB1)    -----...FTRSSKITQAVSSCFT--RPFKFEYS--SGRVGSIYAPEGCPDLCVNLVRGILNSLQITIKK------------------SQNVYELQEAGIGGVCHARYVIQED--
```

Position markers: 222, 229

```
A. mellifera (Q868N5)       FVIHRSPELVPMPTLK-----G--DGRHMEVIKIKNFDNCDQRINYHFGMTDNS---RLEPGTN--KNGKFFSRSSTSRIVIS-ES-LKHF
A. rosae (Q17083)           YVLQTKPELAPVPQLK-----G--SGQIIDIVKTSNFSNCEQRMGYHFGITGET---DWEAGSN--SMGDFLAKSSVSRVVIS-GS-LDRY
P. nipponica (O17428)       FVLQKRPELVPMPDIR-----G--DGEIIDIVKTKNYSNCEQRSGYHFGIPGTN---KWEPSSG--ASGNFLSRSSVSRVIVT-GN-LKSF
P. puparum (B2BD67)         RVVQTNPELVPIPELR-----E--DGDIISLVKTKNYSNCDQRVSYHFGLNGRN---KWEPSGN--GNSKYLSRSSVSRVIVS-GN-LKRY
E. formosa (Q698K6)         EVLQSQPELVPMPELR-----G-DDGEHISIIKTRNFSNTHQRISYHSGISGKN---SFKPGSN--DNGKYLSRSSVSRIVIS-GE-LKKF
B. ignites (B9VUV6)         QNVYVQPELVPVPDLK-----R--EGQYIDIRKSKNFNKCDQRMNYQFGITGNK---YWEAGSN--KNGKFFSQSAMSRIIIS-GT-LKSY
B. hypocrite (C7F9J8)       PNLYVQPELVPVPDLK-----R--EGHYIDIRKSKNFNKCDQRMNYQFGITGNK---YWEAGSN--KNGKFFSQSAMSRIIIS-GT-LKSY
S. invicta (Q7Z1M0)         NF-----DSIPFPNLR-----K--DGLNFFVTKTKNYNKCEQQMAYHSGITDKM---NWKLGSN--D--GVLSRSSTSSIIIS-GN-VKHF
S. invicta (Q2VQM6)         NEIQAKQDRIPMPSLH-----S--DGNHYEVKKLKNYERCQERQLYHYGFDIKSAGKKWA------GQDKVISQLSVTEMVIS-GD-LKRF
R. clavatus (O02024)        YILQARPELAPLPHLK-----G--DGEFIEVVKTRNFSNSNQHVIYRFGSSGHE---GWEAGTN--QMGDFLSRSSQSRVILS-GK-LNRY
A. grandis (Q05808)         YLVQSQPWIAPQYKLK-----G--EGDLIEVIKSKNYTNARDRPSYHYGFGEIE---ESEPTAN--KMGQFFIRQSNSRAILT-GK-PSRY
L. deyrollei (B1B5Z4)       YLLQSKPELAPLPHLR-----A--DGRIIEIVKTRNFSECDQRVAFHFGITGPT---NWEPAGN--QMGPFMARSSVSRVIIS-GK-INKY
A. aegypti (Q16927)         YMIQAHKQWVPQGQLR-----G-EDGQFIQVTKTQNFDRCDQRMGYHFGFTGYS---DFRPNTN--QMGNVASKSLVSYMYLT-GN-WYNF
N. lugens (A7BK94)          RVVQQHPEIAPLAVKQQGQGQGQSHSRLIQVVKSRNFSNCDNPVTYHFGFTQES---NFEPASN--QMGNLVSRAAMGRIIIA-GE-PDSY
G. nigrofuscata (Q9U5F1)    QELMENPKLAPRVGLS-----KLNDIGLFRVVKTINYDNCKLRSAYNFGFNQVP--ASWKPGTN--GMENSPVRSTVTNAIMT-GK-RQRY
A. pernyi (Q9GUX5)          EWRREL------PKFA-----S--QEDPIEITKSKNYGHCHHRVAYHFGVPEGA---EWTGTAHKPNEHQFISRATVFRILAG-KQ-G---
S. japonica (Q59IU3)        EWRREL------PKFA-----S--EEDPIEITKSKNYGHCHHRVAYHFGMPEGA---EWTGTAHNPEEDQFIRRAAVSRILVG-KQ-G---
P. Americana (Q9U8M0)       LPLLQHPELAPLSNVN-----D----NVIDIEKTQNFSNCKRRPAVHYGLAGIP---DLEPGQN--QMGDFLARSSVSRVVIS-GT-LNKF
B. germanica (O76823)       TTYLNP-----WQWTQ-----Q--HDTKLRIMKTHQFTNCRHNSAYKLHFNAFE---YFHLKQH--KPETFLSNSAVSRVIAD-GDNLKNF
R. maderae (Q5TLA5)         TE-MKL-----WNYL---------DNDNLQVTRTQNISHCNS-HLFHLKFSGFE---HFTDRM---NNGGFISNNVVTRMVVD-SV-ENNL
H. vitripennis (Q0ZUC7)     VVLQNKPQLAPMPHLQ-----A--DGQLIDIVKTMNFSNCDVPSAYHFGITGLT---NWEPASN--QMGQFLARSSVSRIIIA-GN-LKRY
I. unicuspis (Q91062)       ---------------------Y--RTNEMAVVKTKDLNNCDHKVYKTMGTAYAE----RCPTC--QKMNKNLRSTAVYNYAIF-DE-PSGY
A. transmontanus (Q90243)   ---------------------R--KANKIIVTKSKDLNNCNEKIKMDIGMAYSH----TCSNC--RKIRKNTRGTAAYTYILK-PT-DTGT
O. aureus (Q9YGK0)          ---------------------A--RAERIHLTKTKDLNHCQEKIMKAIGLEHVE----KCHDC--EARGKSLKGTASYNYIMK-PA-PSGS
O. mykiss (Q92093)          ---------------------A--KAERIHLTKSKDLNNCQQRIMKDFGLAYTE----KCVEC--RQRGEALMGAATYNYLMK-PA-DNGA
F. heteroclitus (Q90508)    ---------------------A--RIENILLTKTRDLSNCQERLNKDIGLAYTE----KCDKC--QEETKNLRGTTTLRYVLK-PV-ADAV
X. laevis (P18709)          ---------------------K--RGDQIRIIKSTDFNNCQDKVSKTIGLELAE----FCHSC--KQLNRVIQGAATYTYKLK-GR-DQGT
G. gallus (P87498)          ---------------------K--KANLVDVTKSKDLNSCEEKVQVVTGSAYTQ----PCQTC--QQRNKNSRATATYNYKIK-YT-HNEA
H. sapiens (P55157)         ---------------------K--------VIKIKALDSCKIARS--------G----FTTPN--QVLGVSSKATSVTTYKVE------DS
A. carolinensis (Q9PUB1)    ---------------------R--KNSRIFVTKTVDSTNCQEKVEKSVGMAYIY----PCPVD--MMKRKAYQRASGFLLQAKAVR-QRAR
```

B) Lipid binding site MSA (residue 426 to 1046)

```
                              427               445 449    577         587   593     602    697 701                           725    769  775
A. mellifera (Q868N5)         KELIFQIANELEDA-----SNIPVHATLEKF...IHNAQVNKRSIHNNYPVHTFGRLTSKHD...SHEVRCTAVFLLMKTNPPLSMLQRMAEFTK-...-EYDYELSRGYIDEKILE
A. rosae (Q17083)             KKLASQIGEELQKP-----SNIPKQNTLEKF...VRESQVNNASAHGRYPVHSFGRLSPKNN...AYQVRTAAVFQLMRTNPPPSMLQRMAEFTN-...-NMGLQYSRGWLHDYTSE
P. nipponica (O17428)         KTLASQIGSDFQDP-----NSITDEQTLEKF...VRYSQMDNPSSHTRYPVHTFGRFSDKIN...AHQLRCAAVTALMSTNPPASMLQRMAEFTN-...-SYGFEYSKLYLTDFIER
P. puparum (B2BD67)           KSVAQEIGNEMEKP-----DLMPETQTLEKV...IRHAMVNQRSAHNRYPVHAFGRLISKDS...HYEIRCAALFTLIKTNPPASMLQRIADFTN-...-KYGPQYSRVVMKTFKNS
E. formosa (Q698K6)           KNIARQIGQELQKP-----DSMVEERTLEKF...IRRAIVNKRSAHNRYPVHVFGRMFNREN...HHEIRTAAVFLLMKTNPPASMLQRMAEFTN-...-SFGPEYSRAVIKEYKNP
B. ignites (B9VUV6)           KELISQIANEMEDP-----NDVYENHILEKY...VYNAQVSRKGLHNHYPVHTYGRLTPKHN...EHQVRCTAVFILMQTDPPLTMLQRMAEFTK-...-DYSYHYSRGYITESIMD
B. hypocrite (C7F9J8)         KELISQIANEMEDP-----NDVYKNHILEKY...VYNAQVNRKGLHNHYPVHTYGRLTPKHD...EHQVRCTAVFILMQTDPPLTMLQRMAEFTK-...-DYSYHYSHGYITESIMD
S. invicta (Q7Z1M0)           ARFIAQIAWNLETSPVNAFNFSPSTEYIEPC...VRYTQSNRS---IHYPVHSFGHMVSKQD...AYELRCAAVYVVMKTNPPLVMLQRMAEFTN-...-IDTESYSQGFLFKKIIP
S. invicta (Q2VQM6)           KNLISEIAEEVQIT------SEGYEATMEKY...LRNSYIG----QNYYPIYSFGFITLKKN...AHEVRAMAVHEFILTDPPMITLQRIAKNTN-...-NYGNWHSSGYHLDFQDW
R. clavatus (O02024)          KSLAKKVGQWVQNP-----GQIPEENRLPLF...SRKSQVDYDVAHNRYPVHLHAKNSQEHR...KSEIRAAAVRQLMRTNPPAQMLQRMADFTN-...-NYGVQYSKTHLKSHNVE
A. grandis (Q05808)           ENLVTEISDEIKQS-----EKTISKHTLDKY...VHKVYINRNESHNQFPVHAFGSFYTKKG...LPEIRVVAVHQLIRANPPVEMLQRMAQYTN-...-QYGMEQSYINLRDYVAE
L. deyrollei (B1B5Z4)         QELAKEIALDVQTP-----NAITGEDTLTKF...VRKAMVNNETLHHRYPVHAYGRLTRKNS...TPEVRVAAIMLIMRTNPPAQILQRMAESTR-...-QYGYHYSKHHMRSYIAE
A. aegypti (Q16927)           GKLVEEIASDLENP-----SDLPKSNTMSKF...VNQAHVSNRSAYNYYPVFSFGRLADADY...VHEVRCAAVHLLMRTDPPADMLQRMAEFTH-...-DVSIQYSFNHIRDYALE
N. lugens (A7BK94)            QKVAMRIGAQVQRP-----SAIPGQNTLTSF...LRKAQVDSDTKHMRFGVHSFGHLTSKHD...AAELRVAALHLLANANPSAAMLQRMAQQTH-...-EYGSSLSKNFLSSFVID
G. nigrofuscata (Q9U5F1)      VKLANEMGEEIVQD----ASSMSGLNTVEKF...LRRTQVNKHIFHSRYPTYTFGPMAAKNN...NHALRCAAVHLLMKTNPSAVLLQRMAQFTN-...-DFGAQYTKNIFHNYISD
A. pernyi (Q9GUX5)            QKLLQDKAQELQNP-----NNMPKSDFLSKL...INMGQVNNDTAHNFYPTHMYGRLARKHD...PYEVRVAAIHNIFIAHPTGAMMQAMAEMTH-...-NFGYQYSFKLFNDGFDM
S. japonica (Q59IU3)          QKLLQDMAQQLQNP-----NNMPKSDFLSKF...INMGQVNNDTAHNFYPTHMYGRLARKYD...PYEVRVAAIHNIFNSHPTVAMMQAMAEMTY-...-NYGYQYSFKLFSDGYDM
P. Americana (Q9U8M0)         KSLAEEIKSDMKKP-----AYIPERSTHAKL...HRQV-HDAEFSHNNYPVHAFGRMVPRNY...NHELRCASVILLMRTQPPAAMLQRMAEFSN-...-SMDIAYSNDILSSNMIQ
B. germanica (O76823)         VRLSKDIAADLQGEP----RVGEDRHILPRF...IHQVEMHQ-----------------VRD...HADIRVEALFLLMKADPSIHVLKMVAELTH-...-NMDVSYSKSYLYGYKSK
R. maderae (Q5TLA5)           VQLANDIGHDLIDPD-----SLPDKDTITKF...YRFIHA-R-----------------LKR...HPDVRVQAVFLLMRSDPSVAVLRTMAELTH-...-NLDVSYSKNYMLDQEAR
H. vitripennis (Q0ZUC7)       KKIAQQIGYEMQSP-----SAIPGQKTLGMF...ARKAFVSNSTAHNRYPVHAFGALKDKNQ...ATEVRVAAFYALMKTNPPAQLLQRSAQFTN-...-VMPAQYSRKNIRSYYNQ
I. unicuspis (Q91062)         KHLVENNQ------------LSVHEDAPAKF...VFRYCANTV-------------------...KSELRIRSCIVFFESKPSVALVSMVAVRLR-...DRLGCRYSKAVHVDTFNA
A. transmontanus (Q90243)     QHLVQNNQ------------QQVQSDAPSKF...VNRYCAETL-------------------...HSEVRMVASMVLLETRPSMALVATLAEALL-...DRLSYRYSKAMHMDTFKY
O. aureus (Q9YGK0)            NHLVASNV------------AKVHEDAPLKF...ISRFCKAQP-------------------...HAELRMVAAMVLFETKLPVGLAASISTALI-...GRLNFRYSRAFHVDTYNN
O. mykiss (Q92093)            NHLVTYNT------------APVHEDAPLKF...VSKYCVEHP-------------------...HPELRMLACIVLFETKPPMGLVITLASILK-...RRLSCHFSQAIHLDAYSN
F. heteroclitus (Q90508)      KHLATYNI------------EDVHENAPLKF...VNKYCNKTV-------------------...HPELRMLSCIVLFETSPSMGLVTTVANSVK-...DRLSLRYSKAVHVDLYNS
X. laevis (P18709)            QHLVQDTQ------------QHIREDAPAKF...VRRYCDQLS-------------------...RTEVRMMACLALFETRPGLATVTAIANVAAR...DNLGYRYSKVMRVDTFKY
G. gallus (P87498)            QDLVETTY------------EQLPSDAPAKA...CHKRCSSPY-------------------...HPRIRMLAAVVLLETKPGLPILMILVDAVL-...DRSGYQFSKVFRFSMFKE
H. sapiens (P55157)           SELWRSTRKYL-QP-----DNLSKAEAVRNF...VRKLCQNEG-------------------...-KTVRTAAAAIILNNNPSYMDVKNILLSIG-...DRF----SRSGSSSAYTG
A. carolinensis (Q9PUB1)      QHIVLNNQ------------QDFHDDVPYRF...VHRYRSQTS-------------------...-------------------------------...------------------
```

```
                                           800     926       936  975          987   994 1000                1035       1045
A. mellifera (Q868N5)         NQN----IITHMILNYVGSEDS...VHSFPTETGLPFV...KNVQGRVGFVTPFEHRHFISGIDSNLHV-YAPLK--...--KSRLFHYSVVPFVSNHD
A. rosae (Q17083)             AMS----SGYQLNSYWIGSEDA...TVSFPTAMGLPFV...SKVIARIGFVNPANHQHHMAGYDKNFQV-NLPLS--...--EYNLFHYSSWPYTAQKD
P. nipponica (O17428)         EMN----VAYQAQASFISSDDS...TMSFPMESGFPFV...VQFQSKLGFVTPFEHQHYMAGINKNYQA-YVPLR--...--KFKLWHHSVVPFTSRHD
P. puparum (B2BD67)           ETN----SGFQLVATYMGSEDS...TISFPMETGYPFT...LKVQKRLGFVIPFEHQEYIAGLDKNIQL-YVPLQ--...--EYKVLQFKSQPFTAKHD
E. formosa (Q698K6)           LTQ----SSVSVEAKYIGGGDS...TVSFPTETGLPFY...MKVQERFGFVVPFENQEYIVGLDKNLQV-HLPIR--...--EVKVLQFKTQPFTAKHD
B. ignites (B9VUV6)           EGN----IISHMMLKSIGSDDS...TYGFPTETGLPFV...MKIQGRMSFVTPFEHQEYIAGVDVDFNL-HLPMK--...--KAQLLHYSVVPYVANHN
B. hypocrite (C7F9J8)         DGN----IISHMMLKYIGSDDS...TYGFPTETGLPFV...MKIQGRMSFVTPFEHQEYIAGIDVDFNL-HLPMK--...--KARLLHYSVVPYVANHN
S. invicta (Q7Z1M0)           SLN----MAEITILQIIGSQDT...TVAFPTESGLPFI...EKVQSRIGFVTPFEHRHYVAGIDTNTRL-VTPLG--...GTGHSTVHFSVVPYTARNN
S. invicta (Q2VQM6)           LVN----G---LSLQT-IAGDD...IMSFANEDGLPFV...NNVQQQFGFVALFEHQKYIAGINNNRVL-RVPVE--...GNELKLLHYSVVPFTTQQD
R. clavatus (O02024)          GQN----LQYQQHCSSIQSQDS...EIGFPSAMGLPFV...AQKSGLMGFVAPFSQNRYIAGLEKNIHA-YVPVQ--...QEEQQVFQYSSNAYTTYQK
A. grandis (Q05808)           QMG----LELHVQRTSHSSAES...ALSFPTEMGLPFL...GKTQSHLSFVTPFDHQIYMAGYDKNMYV-SIPVN--...--DSRLVHITSTPYTSRSD
L. deyrollei (B1B5Z4)         QQN----LAYKHDISLIQSSDS...ELAFPMATGLPFV...TKTQATMGFVTPFNHQRYIAGLDRNIHV-YLPIS--...--YQKLLEYSTVAYTTRHD
A. aegypti (Q16927)           NLE----LSYRLHYGEIASNDH...VLAWPLATGFPFI...RMVDAKVGFVTPFDHQRYIAGYQKKLHG-YLPFN--...--DHLLFHMSSWPYTGYKD
N. lugens (A7BK94)            NID----KSYESQLSSIGSVDS...KVAFPTPMGLPFV...MQSNSKFGMVAPFNHHEYYASVEKNMQF-YMAVQ--...--KQNVFQYSSVLYTTKSN
G. nigrofuscata (Q9U5F1)      EMY----QSYDQYISYIGSEDS...VIAYPMASGLPFF...SKARSRYGFTTPFDHTAYIAAVDKNFQL-SVPR---...--EKNIVHYSVWPYTTPHH
A. pernyi (Q9GUX5)            EDD----LEIFSALSNIGSDDS...SVMFPIAMGVPFI...RNIDGDVGFMDTISNQHVSVGVVSKLQL-NAPVK--...--DYNIVHYSVWPYSAIQK
S. japonica (Q59IU3)          EDE----LEIFNSLSHIGSDDS...SVMFPVAMGVPFI...RNIDGDVGFMDTISNQHVSVGVVSKLQF-NVPIK--...--DITVIHYSVWPYSTIHK
P. Americana (Q9U8M0)         DMD----LGYKDNMAHVGSSDS...AIAFPNALGLPSL...VKEMSKIAIVTPFNSMEHMAALERNILI-NIPIK--...--NPRLAQLSTNTYTTNHK
B. germanica (O76823)         KIN----YDSLYNLNYIGSEDS...TLTLPCAMGLPAY...QMYHAQLAFSTAFDNKEYISGLDRKVEV-HVPVK--...--DYDVLQWQTIPYTTIHN
R. maderae (Q5TLA5)           EYN----LDFQLFYEQIGSQDN...IYGFPTVMGFPGV...AKLQSKLSVITPFNDMRYTAGVNRHFHSRSLPVK--...--DHRVLYMSSIPFTTIHD
H. vitripennis (Q0ZUC7)       QLN----LGYKQAFSYIGSDDG...NVAFPTAMGLPFT...LQAQSSMGFVTPFNHQHYTAGVYKNFQV-NLPIR--...--KQNLFEYSTVPYTSQHS
I. unicuspis (Q91062)         RTM----AGVSADYFRINSPSG...RYMQPVCIGIPMD...QHAVAIMGLTTDLAKAGMQTHYKT--SA-GLGVN--...----VVVLSTMESIVFVR-
A. transmontanus (Q90243)     PLM----AGAAANIHIINNAAS...RRILPTCIGLPME...MHTIAVMGINTHVIQTGVELHVKA--RT-TVPMK--...----EVLSLSAQAFAISRN
O. aureus (Q9YGK0)            AWM----MGAAASAVLINDAAT...RLILPTTVGLPME...MQTYAFMGVNTALIQAAVMTKAKV--YT-AVPAQ--...----TIASAHAETVAIARN
O. mykiss (Q92093)            PLR----IGAAASAFYINDAAT...RRIFPTAVGLPME...MHTFAVMGVNTAFIQAAIMARAKV--RT-IVPAK--...----HIAAAHIETFAVARN
F. heteroclitus (Q90508)      SLA----VGAAATAFYINDAAT...RRILPTVAGIPME...MDTYAVMGLNTDIFQAALVARAKL--HS-VVPAK--...----NITSMNVTTFAVARN
X. laevis (P18709)            NLM----AGAAAKVFIMNSANT...RHIIPTTVGLPAE...VHTVATMGINSPLFQAGIEFHGKV--HA-HLPAK--...----HLVEIRAQTFAFTRN
G. gallus (P87498)            FLM----SGLAAKYFVLNNAGS...RCIVPTCVGFPME...KHMTFVIGINTNMIQAGLEAHTKV--HA-HVPVN--...----NLIIVSSKTFAVTRN
H. sapiens (P55157)           YIERSPRSASTYSLDILYSGSG...QGGLAIDISGAME...VVITTDITVDSSFVKAGLETSTET--EA-GLEFIST...----ERL-STGRGYVSQKR
A. carolinensis (Q9PUB1)      ----------------------...-------------...------------------------------------...-------------------
```

C) Region before the vWF domain MSA (residue 1238 to 1325)

```
                                      1242                                 1279                          1310          1324
A. mellifera (Q868N5)          DYEVCIGSQIM-VSPETLLSYDEKM---DQKPKMDFNVDIRYGKNCGKG-----ERIDMNGKLRQSPRLKELV-GATSIIKDCVEDM-KRGNKILRTCQ
A. rosae (Q17083)              NYQICLSAQSS-RPKVPVMDFIKAL---KADPTTKFAADLKFGEQCQSG-----AQVQIQGQLEQTEGRREYL-RRTPTGQQCAQQM-QEGNKILPACR
P. nipponica (O17428)          EFESCTSLEAR-SPRTS--DFGKDMLDQPEFRSRKFDIDLRYGQTCEKG-----YKVEIQGSQEQTEKYRDTI-KRLPTAIECIRAA-KKGMKSLPACH
P. puparum (B2BD67)            NYHFSAGYEAK-NPDVDTLDFEETL---KANTRREFDAELHYGKGSGDSSDDFKDIIRIQGEAKQSEERKNQI-RQSREAEECNREHNKSGNKMTLACQ
E. formosa (Q698K6)            NHHLSAAVESK-TPNTLSMDFEETL---KSNTPREFDAEVQYGKRSEDSSENSQNKIKIQGKAKQTENRKNEI-RQSRDAQECSKEKSRTGNKMSPACE
B. ignites (B9VUV6)            LFEVCAASQTK-VTPDNTVFYEEVA---QLKPKVEFNADFRIGKVCSTG-----EQLNINSVATQSKELRERI-KNSSLIKTCEKQM-QQGNKILRACQ
B. hypocrite (C7F9J8)          LFEVCAASQTK-VTPDNTVFYEEVA---QLKPKVEFNADFRIGKVCSTG-----EQLNINSVATQSKELRERI-KNSSLIKTCEKQM-QQGNKILRACQ
S. invicta (Q7Z1M0)            IYAELGAIGYV-KSKSISLNPEKAL---EQIPNDEFKAEIRLGNNFNE------EMIKLEGNWTRTDDVKDMA-MKSEIVKKCRQDM-KQGNILLPACQ
S. invicta (Q2VQM6)            NYEVCFSHEMQYAPRATPLNFKYAL---KNSPRDEYKAVLKYGKTCATG-----NKVVITGSSSQSQQLRDII-ENSSLPNNVWKRF-QTGNKAVENCM
R. clavatus (O02024)           PFQAAFQLDIK-DPQLPAFNYQDAL---NADSTSQVQGKINMGEQLE-------SQITFKGKLERTQERKEYL-QQHPLARACESQM-QRRNYIQPACL
A. grandis (Q05808)            -KQGALEIRSE-IPNTNGLNLDDSL---DTEPSTKYNMRLQYGNSENDA-----FEISAQAQLSRSQERKQYLINQDPLYHVCKEQM-QQKNFQLPACQ
L. deyrollei (B1B5Z4)          PFQLTLNVTTD-FPDVPILNYKRAL---RADPTSQVYAHLNFGENAQHG-----AYVYLEGTLEQTKPRIQYV-QRHPLAKLCEQQM-EEGHNILPACR
A. aegypti (Q16927)            QFQFCATATSE-FPRVPYLKFSDFD---KYYGDASQYFDFLYGESCQGG-----AHIAVKGKQKQTGKYREYL-RFSDVAKACKEQM-ANGYYQFEECQ
N. lugens (A7BK94)             KAQVCAAAASN-FPNVPLMNFHDAL---KANPTSRISADIAFGAQCNAG-----GHIHADAKLSQTQEFQEYA-KSRPMAKKCFQLM-EKGQALEYACQ
G. nigrofuscata (Q9U5F1)       PVQVCLAAAAF-MPQVPLVNFIKAL---NANPNSSVNVQISTGHDCTG------ASINIDGKLQQSYERFQYV-RDQPLSRLCINQI-EEGNQLLPACR
A. pernyi (Q9GUX5)             -NQFNGVATVK-LPKSNALNYLQVL---DNDLKTTAEVDIKFNHN---------ANIKIQAEAERSREYTEEL-QKHPLAKQCAQDI-ARNNQYTHTCH
S. japonica (Q59IU3)           -SQFNVVANAK-FPKTNALNFLQVL---DNDLKTTFEADIKFNHN---------GNIHLYAEAERSKKYTEEL-QKYPLAKQCVEDI-ARNNQYTDTCH
P. Americana (Q9U8M0)          EYDLF--AALS-MPAPPIINFNQAL---EFDPDSNLDAGLSIFTNDKPS-----GNLRIKGELQQSEERRNAI-RSTPAALACMREMANGNNNLLPSCR
B. germanica (O76823)          RNQFCLSWSVY-KPQVPIMNIYSAF---EFDPNSKVHAIMNIGKECENGG----SAV-ANIDMLRLSEHLDYV-KNLTVSKLCDHEMRTKRDHVLPACR
R. maderae (Q5TLA5)            NKQVCLSANIM-KPQMPLNNYDDAL---QSDPTSQVRMILNAGNKCQEGS----GQATVDGKLQRTKEYEKFI-KDWALARECQNDMDKYRNYLLRDCQ
H. vitripennis (Q0ZUC7)        MY---VAAESS-MPIVPAMNYKQAL---NADPTSYFNAELAFDD----------AKVQLKGKMQQSQARRQYL-NNYPLAQKCQQQM-QQGNTVLYACR
I. unicuspis (Q91062)          VWKLCAKFRLS--------------------AHMKAKAAIGWGKNCQQY------RAMLEASTGNL---------------------------------
A. transmontanus (Q90243)      RWQICADAILA--------------------SNYKAMALMRWGEECQDY------KVAVSAVTGRL---------------------------------
O. aureus (Q9YGK0)             NWRICADIVQL--------------------SSHKLMAKTAWGAECKQY------STMIVAETGLL---------------------------------
O. mykiss (Q92093)             NWKLCADGVLL--------------------SKHKVTAKIAWGAECKDY------NTFITAETGLV---------------------------------
F. heteroclitus (Q90508)       NWRICADAVVL--------------------SKHKVTTKISWGEQCRKY------STNVTGETGIV---------------------------------
X. laevis (P18709)             RWAACFDAVVV--------------------NPHEAQASLKWGQNCQDY------KINMKAETGNF---------------------------------
G. gallus (P87498)             NWKACADAVIL---------------------PLKAQARMRWGKECRDY------RIAALATTGQM---------------------------------
H. sapiens (P55157)            ---------------------------------------------------------------------------------------------------
A. carolinensis (Q9PUB1)       ---------------------------------------------------------------------------------------------------
```

D) C-terminal region MSA (residue 1648 to 1770)

```
                                 1650                      1687    1696  1702      1711 1715         1729                                      1768
A. mellifera (Q868N5)          HDCIRQERTQQRNVISDS--...--NKHC-TIHRTQVKETDDKICFTMRPVVSCASG-CTAVETKSKPYKFHCMEKNEA-AMKLKKRIEKGANPDLSQKPVSTT--EELTVPFVCKA
A. rosae (Q17083)              AHCYRE-AVLFGNVVSDQ--...KKSGSC-TKFATATIEENGKTCFSLRPVATCAAG-CKATRKQETTVKMHCVQSSSS-SQQLVHRIKQGANPDFSQKSHDKS--SRIEVPESCVA
P. nipponica (O17428)          AVCSQK-SPRPGNVVSDR--...SDHKRC-NTLRTKVIEEEDQICFSLRPLPTCAEG-CTANRTKPKVMPMHCMPKNIA-AERMADRIKQGANPDFSQKSYTKK--NGFDIPVGCHA
P. puparum (B2BD67)            SQCTFE-STRPGNVISDT--...RESRRC-MTHRTKVVMKNNQICFSLRPLPTCSSS-CRSAETKPRAVQFHCVARSSA-SEKVAERVEKGANPDLTQKSVSYT--ETYQLPISCRA
E. formosa (Q698K6)            SPRVSL-SARPGNVISDR--...ESEKQC-MKHRTMVQRNKDQICFSKRPVPSCSSR-CSEAETRNKKIEFHCVPKSDA-SEKVADRVEKGANPDLTQKNTSKT--EFQKIPVSCKA
B. ignites (B9VUV6)            QDCVYEEKNHLSNVISSK--...-KSKQC-TIMKAHMKEIEHMICFTIRQVPSCAPG-CWATEMKPKDYQYHCMKRNAA-SLALKARIEKGAKPDLSQKSVTLT--EPINVPLACKA
B. hypocrite (C7F9J8)          QDCVYEEKNHLSNVISDK--...-KSKQC-TIMRTRIKETGNMICFTIRQVPSCAPG-CWATEMKSKDYQYHCMKRNAA-SLALKARIEKGAKPDLSQKSVTLT--EPINVPLACKA
S. invicta (Q7Z1M0)            STCMSR-SYRPSDVISDR--...----------------------------------------------------------------------------------------------
S. invicta (Q2VQM6)            STCHEFPENEQMNVISDR--...DKKKHN-IVYRTRVVEEGDEICFTTTPLPACRQG-ARPTERYPKKADLYCMPRNDQ-SLDLKRRVEDGANPDFTRKSVSRM--QVFQVPVSCSA
R. clavatus (O02024)           SPSYER-RVILGDVVNEL--...VQGSSC-SKLMTQIVEENDKSCFSTTPQPACASH-CRPTGKIQKIVDFHCVQASSS-SRNWAQMIKNGANPDFSAKEKHRQ--ILLQLPTGCVP
A. grandis (Q05808)            EQCIKKVLPLYTNVISNQ--...-TKLASGTVMKHRYIEENGEICFTIRPLPVCNTS-VK--QVVTKNVPVHCIQGTKT-AYYYKSLIDQGGNPDFSRKSETRT--ARMEVAAQCN-
L. deyrollei (B1B5Z4)          -PCYEP-EVILGEVVSER--...DSKRAC-THYRVKVLEVGKKMCFSLRAHIACGAG-CQPASKLEKKVDFHCIENSAA-AQHWADSIAKGHNPDFSKKQPNYR-AT-VRLPENA--
A. aegypti (Q16927)            QECVKR-EVYYGDIIYNQEY...HSMKECPVQHQHQFFEQGDRTCFSLRPLPVCHSK-CVATEKISKYFDVHCFEKDSTQAKKYKSDIGRGYTPDFKSFAPHKT--YKFNYPKSCVY
N. lugens (A7BK94)             MTCFRR-RHIFANVITSN--...SERLANPTKLIQDVKNNGDQVCISIRPVPKCQKG-FSPAGSSEKEVDYVCMSHGKN-AQFWINQIFQGGYVLLEQKQHNAT--FMKNIPQRCVR
G. nigrofuscata (Q9U5F1)       ITCVKK-NDKHHNIIPSF--...HGPQSC-TNMRIMIQKRGSRTCFSAQPQNVCSSK-CQATEIIQKEVPFRCVENEKA-RDFIQPVMRGRVPPKLNNYQPSEK--LVIGVPQSCIF
A. pernyi (Q9GUX5)             QEAYQP-ENKYTSVLRSD--...KQRGPCAIKQQVQYYENHGEICITTTQLPACQSH-CRGEDYKIQAAQVTCRPKIDQQFRSHRDHIKQGQNPEVTGVPKVKQ----FKVPTACNA
S. japonica (Q59IU3)           QEAYQP-KNKYTTVLRSD--...KQRGPYVVKHQVQYYENHGEICITTTQLPVCQSH-CTVMEYRIQATQVTCRPKLDQQFRSYRDQIKQGQGPVVTGVPKVKQ----FKVPAICNA
P. Americana (Q9U8M0)          QPCVSR-NFTSTDIIGKHMP...SPPQTS-TLHRTMVVEEVTEICFSMRPLPECAPR-FKPADTLKKKIKFHCLTKGPT-ASHWLKMVKKGVNPDFSKKREHKQ--LEVDIPAKCVR
B. germanica (O76823)          QTCVPQFHQPLVS-------...TSDDKC-MTQQPRHTYYENQFCVSEKPLDTCMPLICHATESYTIDVNFYCVPLGPA-ANHYMKLVKKGILPDLSRNRNGKRVVLPVEIPIQCEP
R. maderae (Q5TLA5)            KTCIRYFPIDDMNYFPKQQR...IHRKQN-MVVITRVVRRDTDVCFSAEPLKTCIDNSRA-ADTRIQQQQFICLPDSPA-SEHYLKLIKKGINPDFTRKKNF--VQLEVKIPTKCIK
H. vitripennis (Q0ZUC7)        GHYSRN-VVIYGNVVTDA--...EKYVSC-SQRRLMTMHKDGKQCFSVQPQLHCTDQ-CSPQGYVNKEVQFMCVLTSSV-SEHWKKLVNRGINPDFSDKHAYFQ-TYNVQVPQSCQA
I. unicuspis (Q91062)          -ACALS--------------...------VHKESTSVISGSRENCYSTEPIMRCPAT-CSASRSVPVSVAMHCLPAESE-AIS-LAMSEG-RPFSLSGKSEDLV--TEMEAHVSCVA
A. transmontanus (Q90243)      -KTAT---------------...----------------EAASFCVIM---------------------------------------------------------------------
O. aureus (Q9YGK0)             -ECSIK--------------...------VKLEKRVIFEGVESKCYSVEPVLQCLPG-CIPVRTTTVNVGFHCLPSDTT-V----DR-SGL--SSFFEKSIDLR--DTAEAHLACRC
O. mykiss (Q92093)             -ECLMK--------------...------VKLEKQVIVDDRESKCYSVEPVLRCLPG-CLPVRTTPITIGFHCLPVDSN-L----NRSEGL--SSIYEKSVDLM--EKAEAHVACRC
F. heteroclitus (Q90508)       -ECRLK--------------...------VQLEKQLTIHGEDSTCFSVEPVPRCLPG-CLPVKTTPVTVGFSCLASDPQ--------------TSVYDRSVDLR--QTTQAHLACSC
X. laevis (P18709)             -GCNLK--------------...------VKLEKAIATDGAKAKCYSVQPVLRCAKG-CSPVKTVEVSTGFHCLPSDVS-L----DLPEGQIRL---EKSEDFS--EKVEAHTACSC
G. gallus (P87498)             -GCKLQ--------------...------VKLNRNPTIDGEESTCYSVDPVLKCMKD-CTPIEKTSVKVGFHCFPKATA-V----SLLEWQRSSDKKSASEDVV--ESVDADIDCTC
H. sapiens (P55157)            --------------------...----------------------------------------------------------------------------------------------
A. carolinensis (Q9PUB1)       --------------------...----------------------------------------------------------------------------------------------
```

Figure S2

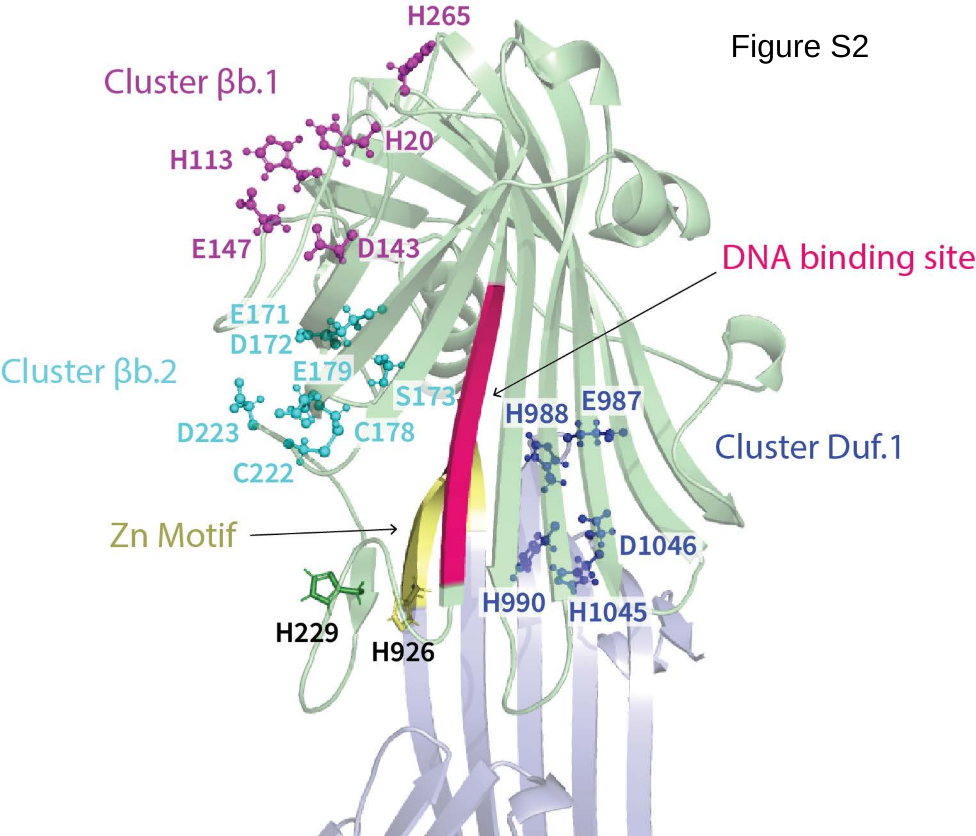
H265
Cluster βb.1
H20
H113
E147
D143
DNA binding site
E171
D172
Cluster βb.2
E179
S173
H988
E987
D223
C178
Cluster Duf.1
C222
Zn Motif
D1046
H229
H926
H990
H1045

Figure S3

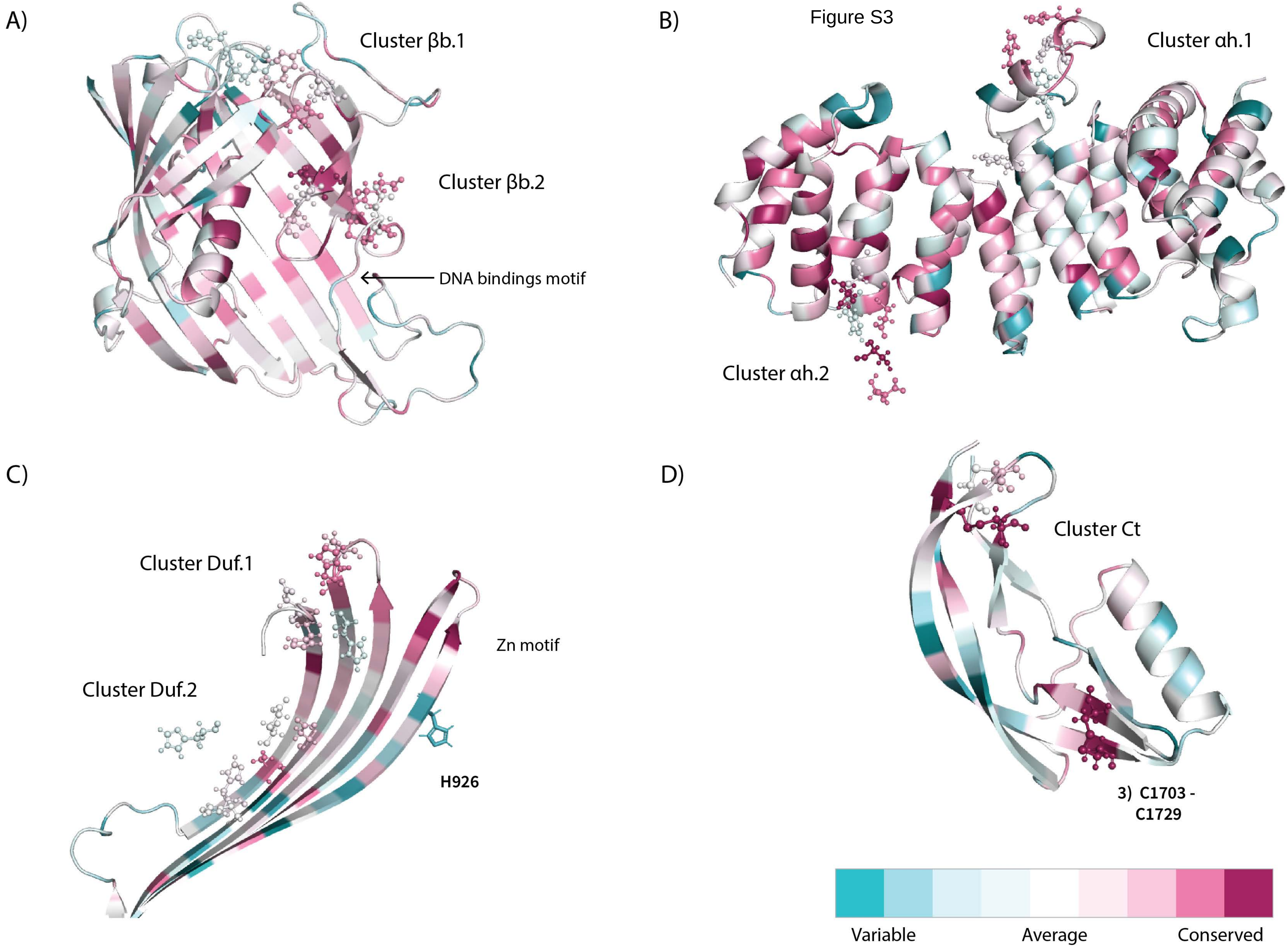
A)
Cluster βb.1
Cluster βb.2
DNA bindings motif
B)
Cluster αh.1
Cluster αh.2
C)
Cluster Duf.1
Cluster Duf.2
Zn motif
H926
D)
Cluster Ct
3) C1703 - C1729
Variable
Average
Conserved

Figure S4

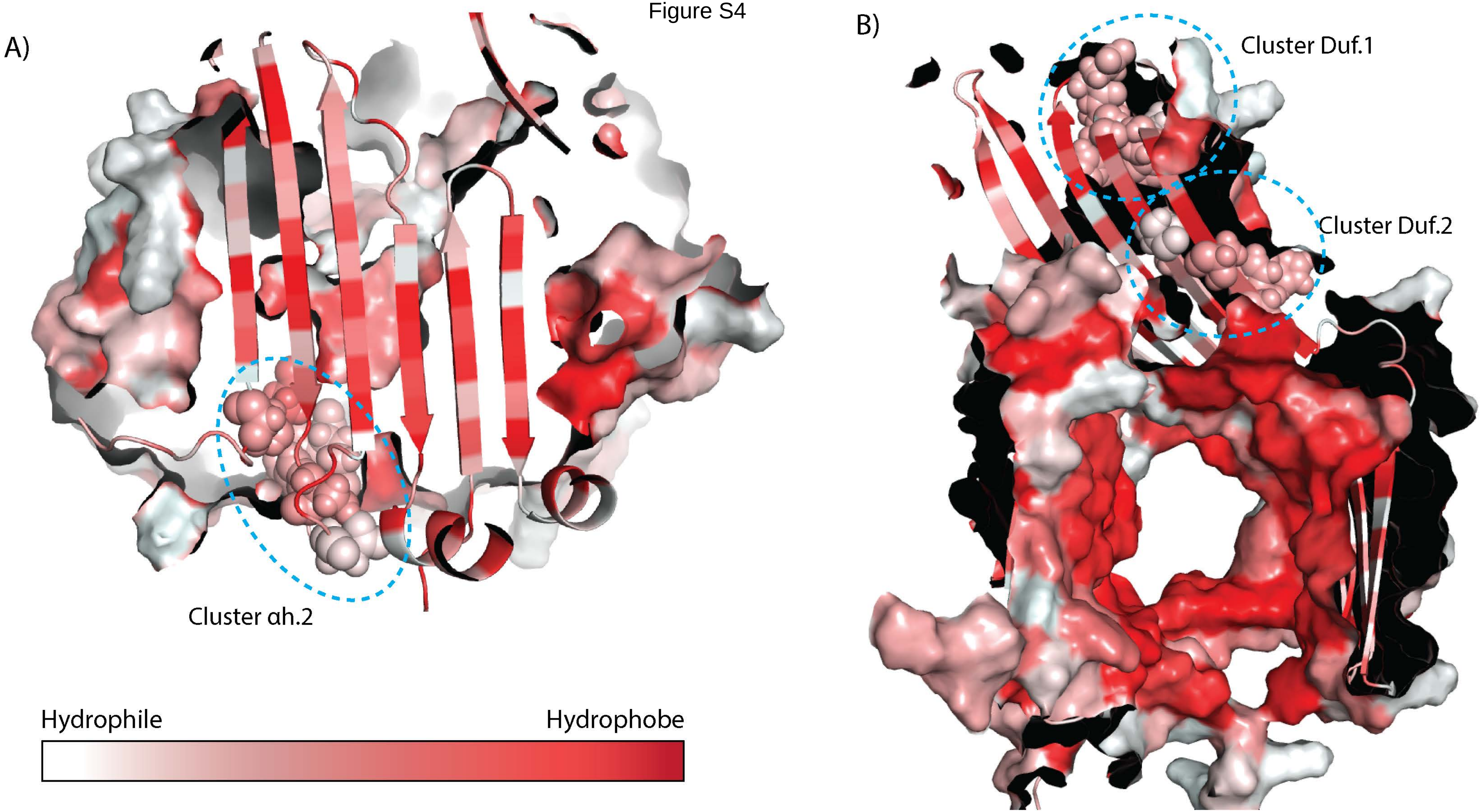

A)
B)
Cluster Duf.1
Cluster Duf.2
Cluster αh.2
Hydrophile
Hydrophobe

Figure S5

A

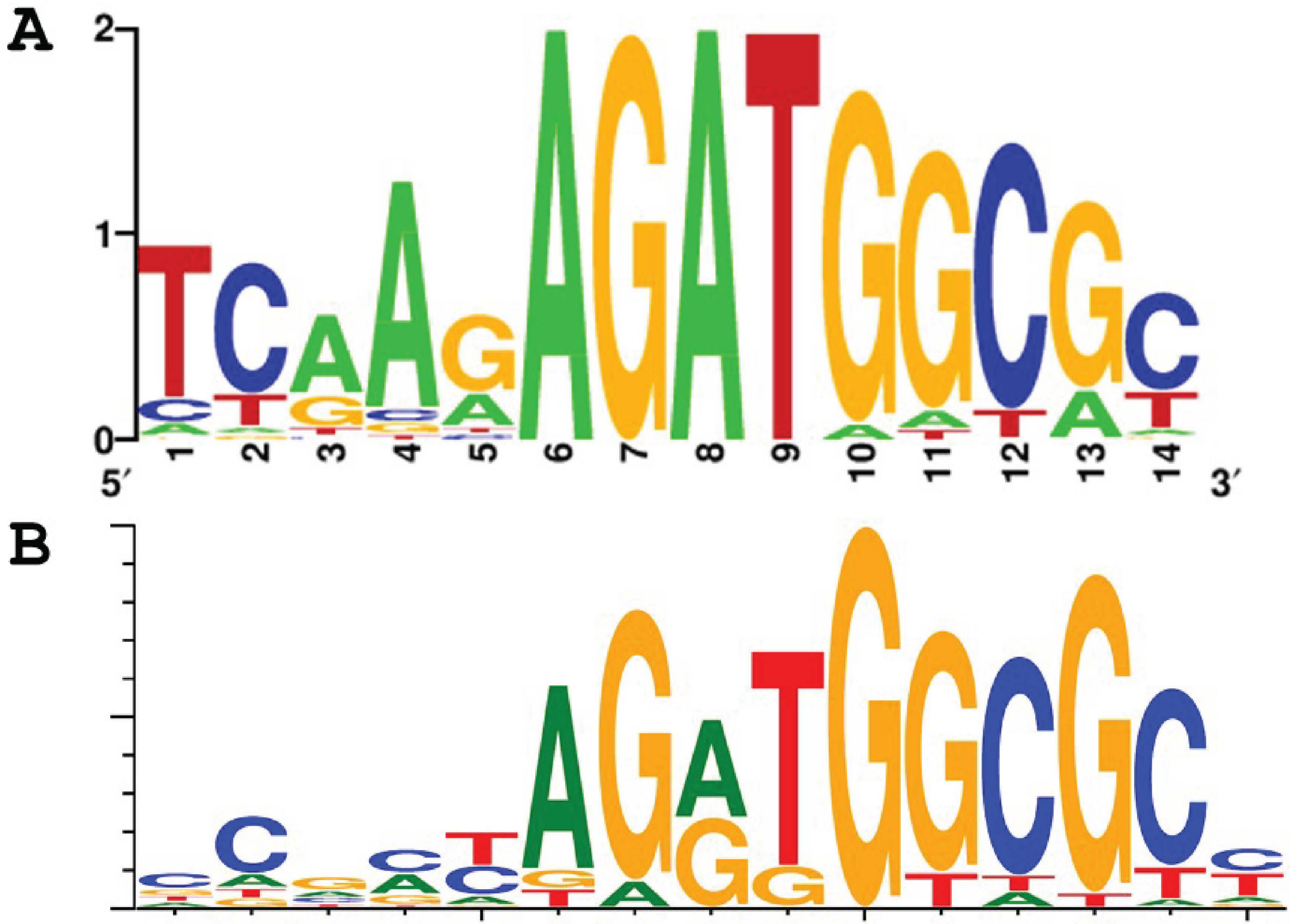

C

```
                                   143 147                               178                                                                            222     229
       A. mellifera (Q868N5)        LQVDTQ----GENAVKVNS--VQVPTDD--EPYASFKAMEDSVGG----KCE-VLY-DIAPL-SDFVIHRSPELVPMP-T-----LKG-DG------RHMEVIKIKNF---------DNCDQ--RINYHFG
           A. rosae (Q17083)        LQVDTQ----GENAMKSRY--NQEPEGD--QAYATYKAMEDTVTG----ECE-VLY-DISPL-PEYVLQTKPELAPVP-Q-----LKG-SG------QIIDIVKTSNF---------SNCEQ--RMGYHFG
       P. nipponica (O17428)        LQVDTQ----GENLMASKY--NQEPEDE--GVTAMFKTMEDTVGG----RCE-VLY-DINPL-PEFVLQKRPELVPMP-D-----IRG-DG------EIIDIVKTKNY---------SNCEQ--RSGYHFG
        P. puparum (B2BD67)         LQVDTQ----GENAIRSKH--NQFPEGK--QPYALFKVMEDSVGG----MCE-VLY-DVSAL-PDRVVQTNPELVPIP-E-----LRE-DG------DIISLVKTKNY---------SNCDQ--RVSYHFG
        E. formosa (Q698K6)         LQVDAR----GQNAEYSKH--NQLPEGK--QPYAIFKTREDSVSG----RSE-VTY-DISPL-PEEVLQSQPELVPMP-E-----LRGDDG------EHISIIKTRNF---------SNTHQ--RISYHSG
        B. ignites (B9VUV6)         LQVDSL----GENAIRTSE--MQIPTDE--HPYGMFRAMEDSVGG----KCE-VLY-DITPL-PEQNVYVQPELVPVP-D-----LKR-EG------QYIDIRKSKNF---------NKCDQ--RMNYQFG
      B. hypocrite (C7F9J8)         LQVDSL----GENAIRTSE--TQIPTDE--YPYGMFRAMEDSVGG----KCE-VLY-DIMPL-PEPNLYVQPELVPVP-D-----LKR-EG------HYIDIRKSKNF---------NKCDQ--RMNYQFG
        S. invicta (Q7Z1M0)         LQVDLQ----GENVIASSD--NQIPDDS--QPFGVYKAMEDSVGG----KCE-VLY-SITPV-PENF-----DSIPFP-N-----LRK-DGL-----NFF-VTKTKNY---------NKCEQ--QMAYHSG
        S. invicta (Q2VQM6)         LQVDTQ----GENAINSKS--IQVPSDE--SFAATFKAMEDSVSG----KCE-VLY-EITPL-TVNEIQAKQDRIPMP-S-----LHS-DG------NHYEVKKLKNY---------ERCQE--RQLYHYG
       R. clavatus (O02024)         FQVDTQ----GKNLKDSKI--NLVPSGQSGEPMGVYKTMEDSVNG----IYE-TLY-DISVL-PEYILQARPELAPLP-H-----LKG-DG------EFIEVVKTRNF---------SNSNQ--HVIYRFG
        A. grandis (Q05808)         FQMDLQ----GENALQNPT--SSFPTNE--YMDAVFKTMEETVTG----KTE-TIY-DIHRL-PEYLVQSQPWIAPQY-K-----LKG-EG------DLIEVIKSKNY---------TNARD--RPSYHYG
       L. deyrollei (B1B5Z4)        LQIDTA----ARNLQKSRI--NQLPAMH--KPMGVYKTMEDTVTG----ECE-TVY-DVSPL-PDYLLQSKPELAPLP-H-----LRA-DG------RIIEIVKTRNF---------SECDQ--RVAFHFG
        A. aegypti (Q16927)         LQVDTR----GANLMHSSK--PIHPSKN--EWNGHYKVMEPLVTG----ECE-THY-DVNLI-PAYMIQAHKQWVPQG-Q-----LRGEDG------QFIQVTKTQNF---------DRCDQ--RMGYHFG
         N. lugens (A7BK94)         FQVDVT----GQNAIKSRR--NIVPNGQ--QVSGSFKVMEDSVTG----KCE-THY-DVDEL-PMRVVQQHPEIAPLAVK-----QQG-QGQGQSHSRLIQVVKSRNF---------SNCDN--PVTYHFG
   G. nigrofuscata (Q9U5F1)         LQFDTE----GENIQKSRS-NSLFDNSD--DPVVTFKTMEASVTG----KCE-TLY-DFSPL-LKQELMENPKLAPRV-G-----LSKLNDI-----GLFRVVKTINY---------DNCKL--RSAYHFG
         A. pernyi (Q9GUX5)         LQLDLS----THRNIHGSQ--DSY-DRE--QKQGLFRKMETDVTG----DCE-TLY-SVSPV-------ASEWRRELP-K-----FAS-QE------DPIEITKSKNY---------GHCHH--RVAYHFG
       S. japonica (Q59IU3)         LQLDLS----THRNIQGSQ--NRY-DQE--QKQGLFRKMETDVTG----DCE-TLY-TVSPV-------ASEWRRELP-K-----FAS-EE------DPIEITKSKNY---------GHCHH--RVAYHFG
      P. Americana (Q9U8M0)         LQVNTQ----ADNLVRHRY--NILPNAS--TDSAVYSIRETTITG----ECE-VEY-DVSPL-PALPLLQHPELAPLS-N-----VND---------NVIDIEKTQNF---------SNCKR--RPAVHYG
      B. germanica (O76823)         LQVVVD---------EDKK---------------VYRVFESTVTG----RCE-ALY-EVDHLYPTTYL--NPWQWT---Q------QH-D-------TKLRIMKTHQF---------TNCRH--NSAYKLH
        R. maderae (Q5TLA5)         IQLTTQ------------------PSFK---P--VYRVKESIITG----RCH-TLY-DFSPL-----LKTEMKLWNY--------LDN---------DNLQVTRTQNI---------SHCNS--HL-FHLK
     H. vitripennis (Q0ZUC7)        FQVDTQ----AENLQKSRI--NSLPTQE--TVNGVYKTMEDSVSG----ECE-TLY-DISPL-PKVVLQNKPQLAPMP-H-----LQA-DG------QLIDIVKTMNF---------SNCDV--PSAYHFG
       I. unicuspis (Q91062)        FQLSLK---------KNQQ---------------TFELQETGVEG----ICQ-TTY-VVQE-------------------G-----YRT---------NEMAVVKTKDL---------NNCDH--KVYKTMG
   A. transmontanus (Q90243)        LQLTIK---------TTQN---------------VYGLQENGIAG----ICE-ASY-VIQE-------------------D-----RKA---------NKIIVTKSKDL---------NNCNE--KIKMDIG
          O. aureus (Q9YGK0)        LQLNVK---------KTQN---------------VYEMQESGAHG----VCK-TNY-VIRE-------------------D-----ARA---------ERIHLTKTKDL---------NHCQE--KIMKAIG
          O. mykiss (Q92093)        LQLNIK---------KTQN---------------VYELQEAGAQG----VCK-THY-VIRE-------------------D-----AKA---------ERIHLTKSKDL---------NNCQQ--RIMKDFG
    F. heteroclitus (Q90508)        LQLNIK---------KTHK---------------VYDLQEVGTQG----VCK-TLY-SISE-------------------D-----ARI---------ENILLTKTRDL---------SNCQE--RLNKDIG
          X. laevis (P18709)        LQVTIK---------KSQD---------------VYDLQESSVGG----ICH-TRY-VIQE-------------------D-----KRG---------DQIRIIKSTDF---------NNCQD--KVSKTIG
          G. gallus (P87498)        LELSLK---------KMQH---------------SYSIQEAGIGG----ICN-TTY-AIQE-------------------N-----KKA---------NLVDVTKSKDL---------NSCEE--KVQVVTG
         H. sapiens (P55157)        FQTQLS----------SGT------------------TNEVDISG----NCK-VTY-QAHQ-------------------D------K---------------VIKIKAL---------DSC----KIARS-G
     A. carolinensis (Q9PUB1)       LQITIK---------KSQN---------------VYELQEAGIGG----VCH-ARY-VIQE-------------------D-----RKN---------SRIFVTKTVDS---------TNCQE--KVEKSVG
     D. melanogaster (Q9VS55)       LRVHIKHMHTSDVPMTCRRCGQQLPDRY------QYKLHVKSHEGEKCYSCKLCSYASVTQRHLASHMLIHLDEKPFHCDQCPQAFRQ-RQLLR---RHMNLVHNEEYQPPEPREKLHKCPSCPREFTHKG
          A. gambiae (Q4G266)       LRIHVQNLHTADKPIKCKRCDSTFPDRY------SYKMHAKTHEGEKCYRCEYCPYASISMRHLESHLLLHTDQKPYKCDQCAQTFRQ-KQLLK---RHMNYYHNPDYVAPTPKAKTHICPTCKRPFRHKG
            B. mori (H9IXV8)        LRIHVQKLHTSDKPLKCKRCGKTFPDRY------SCKMHNKTHNGEKCYKCELCPYASTTLRHLKSHMLKHTDEKPFSCDQCDQSFRQ-KQLLR---RHQNLYHNPHYVAKPPKEKTHSCHECKRNFAHKG
      T. castaneum (D6WGY1)         LRIHVQKLHTSDKPLKCKRCGKSFPDRY------SYKVHNKTHEGEKCYKCDLCPYASISARHLESHMLIHTDQKPYHCDQCDQSFRQ-KQLLK---RHQNLYHNPDYIPPPPREKTHECPECSRAFRHKG
   A. mellifera (A0A7M7MWC7)        LRIHVQKLHTSDKPLKCKRCGKTFPDRY------SYKLHSKTHEGEKCYKCDLCPYASISARHLESHMLIHTDQKPYQCDHCFQSFRQ-KQLLK---RHCNLYHNPSYVPPPPQEKTHQCPECERPFRHKG
   A. mellifera (A0A7M7MWF4)        LRIHVQKLHTSDKPLKCKRCGKTFPDRY------SYKLHSKTHEGEKCYKCDLCPYASISARHLESHMLIHTDQKPYQCDHCFQSFRQ-KQLLK---RHCNLYHNPSYVPPPPQEKTHQCPECERPFRHKG
  N. vitripennis (A0A7M7H6S9)       LRIHVQKLHTSDKPLKCKRCGKTFPDRY------SYKLHSKTHEGEKCYKCDLCPYASISARHLESHMLIHTDQKPYQCDHCYQSFRQ-KQLLK---RHCNLYHNPNYVPPPPQEKTHQCPECERPFRHKG
```

Figure S6

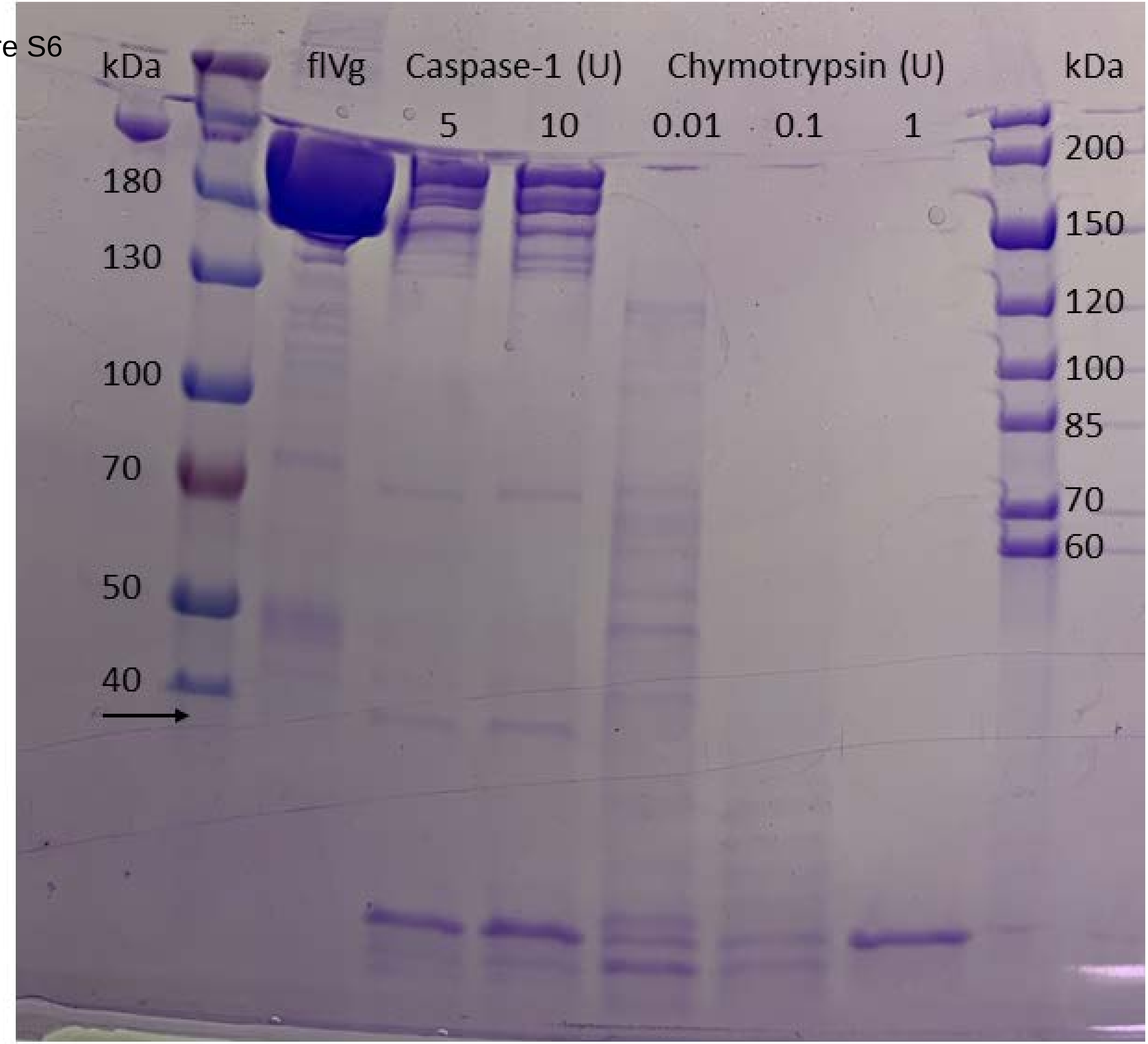
kDa
flVg
Caspase-1 (U)
Chymotrypsin (U)
kDa
5
10
0.01
0.1
1
180
130
100
70
50
40
200
150
120
100
85
70
60

Figure S7

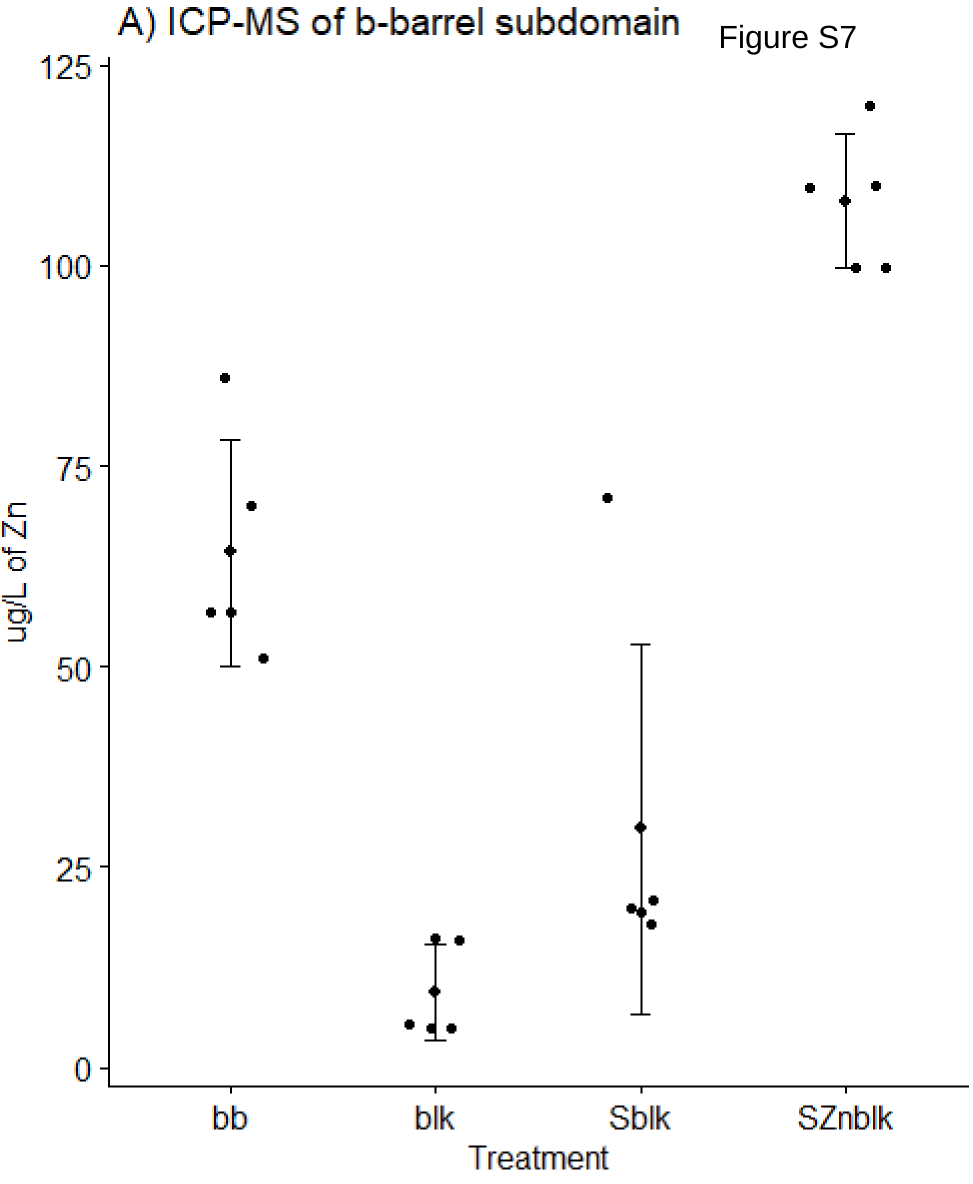
A) ICP-MS of b-barrel subdomain
125
100
75
50
25
0
ug/L of Zn
bb
blk
Sblk
SZnblk
Treatment

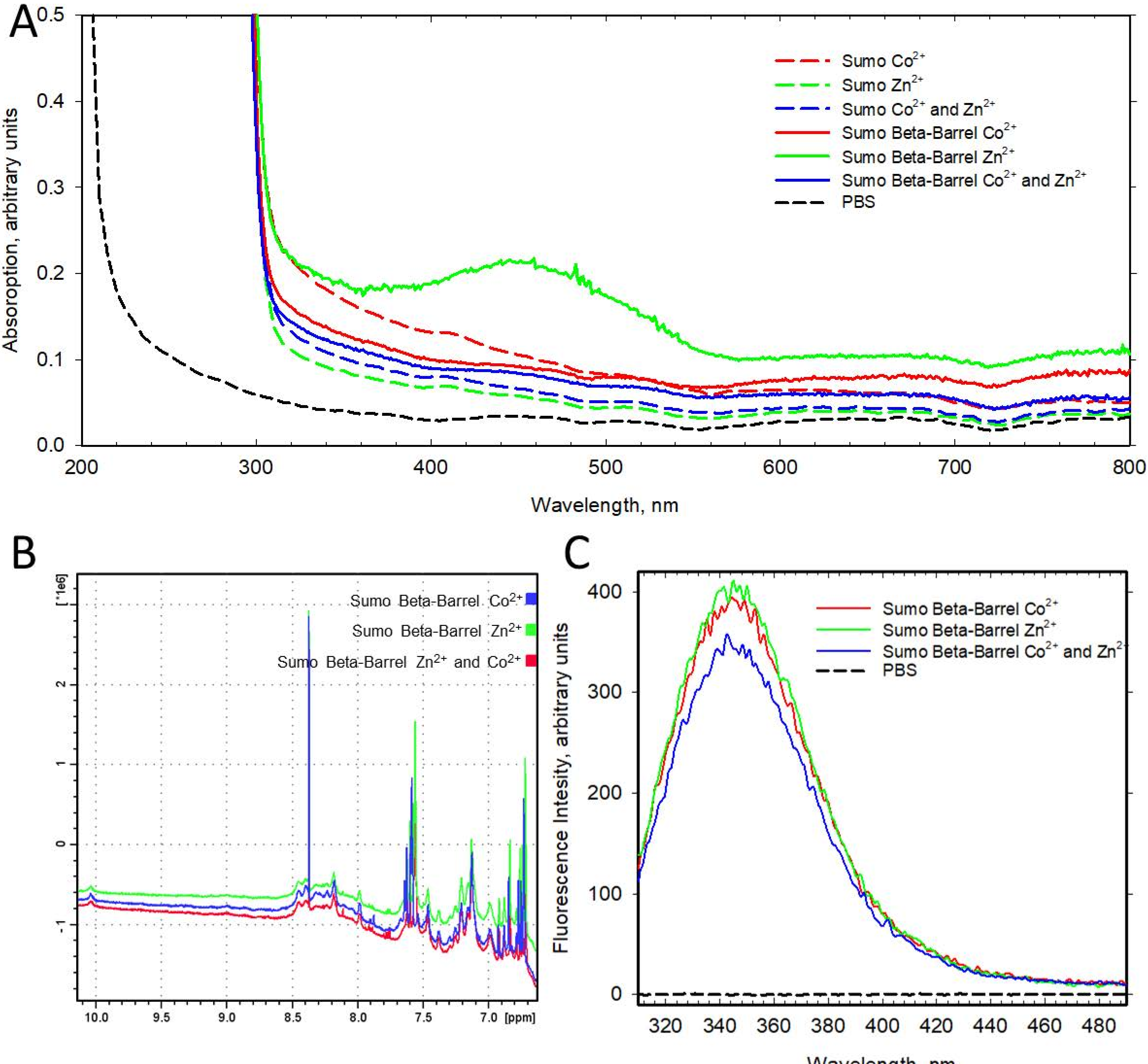
A
0.5
0.4
0.3
0.2
0.1
0.0
Absoroption, arbitrary units
200
300
400
500
600
700
800
Wavelength, nm
Sumo Co2+
Sumo Zn2+
Sumo Co2+ and Zn2+
Sumo Beta-Barrel Co2+
Sumo Beta-Barrel Zn2+
Sumo Beta-Barrel Co2+ and Zn2+
PBS
B
[*1e6]
2
1
0
-1
10.0
9.5
9.0
8.5
8.0
7.5
7.0 [ppm]
Sumo Beta-Barrel Co2+
Sumo Beta-Barrel Zn2+
Sumo Beta-Barrel Zn2+ and Co2+
C
400
300
200
100
0
Fluorescence Intesity, arbitrary units
320
340
360
380
400
420
440
460
480
Wavelength, nm
Sumo Beta-Barrel Co2+
Sumo Beta-Barrel Zn2+
Sumo Beta-Barrel Co2+ and Zn2+
PBS

Figure S8

Measured protein concentration

Sample	Qubit		Mass		Molar mass of Vg	
Vg1	1.460	µg/µl	73.0	µg	0.000363	µmol
Vg2	1.260	µg/µl	63.0	µg	0.000313	µmol
Vg3	1.390	µg/µl	69.5	µg	0.000346	µmol
Vg4	1.280	µg/µl	64.0	µg	0.000318	µmol
Vg5	2.280	µg/µl	114.0	µg	0.000567	µmol
Vg mean	1.534	µg/µl	76.7	µg	0.000381	µmol
Blank1	0	µg/µl	0	µg	0	µmol
Blank2	0	µg/µl	0	µg	0	µmol
Blank3	0	µg/µl	0	µg	0	µmol
Blank4	0	µg/µl	0	µg	0	µmol
Blank5	0	µg/µl	0	µg	0	µmol
Blank mean	0	µg/µl	0	µg	0	µmol

Sample volume	50	µl
Vg molar mass	201147.70	g/mol

Measured element concentration

Sample	ICP-MS*		Mass		Molar mass of Zn	
Vg1	1230	µg/L	0.0615	µg	0.000941	µmol
Vg2	1230	µg/L	0.0615	µg	0.000941	µmol
Vg3	1630	µg/L	0.0815	µg	0.001247	µmol
Vg4	1620	µg/L	0.0810	µg	0.001239	µmol
Vg5	1910	µg/L	0.0955	µg	0.001461	µmol
Vg mean	1524	µg/L	0.0762	µg	0.001165	µmol
Blank1	17	µg/L	0.00085	µg	0.000013	µmol
Blank2	17	µg/L	0.00085	µg	0.000013	µmol
Blank3	17	µg/L	0.00085	µg	0.000013	µmol
Blank4	17	µg/L	0.00085	µg	0.000013	µmol
Blank5	17	µg/L	0.00085	µg	0.000013	µmol
Blank mean	17	µg/L	0.00085	µg	0.000013	µmol

Sample volume	0.00005	L
Zn molar mass	65.38	g/mol

All ICP-MS results in w/V		
Limit of detection, LOD in w/V	5	µg/L
Limit of quantification, LOQ in w/V	17	µg/L
* multipled by 5 to adjust for the five-fold dilution		

Ratio Calculation

Sample	Zn:Vg (Minus the Zn molar mass in the blanks)
Vg1	2.556102
Vg2	2.961832
Vg3	3.570177
Vg4	3.852953
Vg5	2.554382
Vg mean	3.022443
